# Integrating multiple omics approaches to identify AIF1 as a protective factor in IgA nephropathy

**DOI:** 10.1016/j.jtauto.2026.100407

**Published:** 2026-10-07

**Authors:** Xingyu Liu, Yiyang Hu, Wenjia Zhang, Chaohong Nie, Fengxia Zhang, Yufeng Liu, Xiaoyan Su, Shuangxin Liu, Quhuan Li

**Affiliations:** a School of Bioscience and Bioengineering, South China University of Technology, Guangzhou, 510006, China; b Guangdong Provincial People's Hospital, Guangdong Academy of Medical Sciences, Southern Medical University, Guangzhou, 510080, China; c Guangdong Geriatric Institute, Guangdong Provincial People's Hospital, Guangdong Academy of Medical Sciences, Southern Medical University, Guangzhou, 510080, China; d Guangdong Cardiovascular Institute, Guangdong Provincial People's Hospital, Guangdong Academy of Medical Sciences, Southern Medical University, Guangzhou, 510080, China; e The First Clinical Medical School of Gannan Medical University, Ganzhou, Jiangxi Province, 341000, China; f Department of Nephropathy, Dongguan Tungwah Hospital, Dongguan, Guangdong Province, 523015, China; g Department of Nephrology, Guangdong Provincial People's Hospital, Guangdong Academy of Medical Sciences, Southern Medical University, Guangzhou, 510080, China

**Keywords:** IgA nephropathy, Mendelian randomization, Plasma proteomics, Proteome-wide association study, Transcriptome-wide association study, Single-cell transcriptome-wide association study

## Abstract

IgA nephropathy (IgAN) is the most prevalent primary glomerular disease worldwide, and its diagnosis still relies on invasive kidney biopsy. To identify noninvasive biomarkers and therapeutic targets, we applied a multi-ancestry Mendelian randomization (MR) framework using proteome-wide association data from an Icelandic population, with validation in independent Fenland and United Kingdom cohorts, followed by transcriptomic, single-cell RNA sequencing (scRNA-seq), and cell–cell communication analyses. Nineteen candidate proteins were identified, and Bayesian colocalization supported a shared genomic region for eight, including AIF1. Transcriptomic analysis revealed downregulation of AIF1 in peripheral blood mononuclear cells from patients with IgAN, and scRNA-seq localized this depletion to dendritic cells (DCs), with partial restoration 150 days after tonsillectomy. Clinical phenotype MR showed inverse associations between AIF1 and hematuria and albuminuria, and enzyme-linked immunosorbent assay (ELISA) showed reduced circulating AIF1 in patients. AIF1 may protect against IgAN through DC-mediated antigen presentation, representing a candidate noninvasive biomarker and therapeutic target.

## 1 Introduction

Immunoglobulin A (IgA) nephropathy (IgAN) is the most common primary glomerular disease worldwide. It is characterized by the deposition of IgA-formed immune complexes in the mesangial regions of the kidney. The disease has a spectrum of clinical manifestations, ranging from asymptomatic hematuria and proteinuria to progressive impairment of kidney function, ultimately leading to kidney failure. The pathophysiology of IgAN is complex and multifactorial, and involves genetic predisposition, environmental triggers, and dysregulated immune responses that contribute to aberrant IgA production and subsequent glomerular deposition [1,2]. The systemic immune environment in the blood acts as a crucial upstream trigger that precipitates the subsequent local kidney tissue injury [3,4].

Despite advances in the understanding of the disease, current diagnostic strategies for IgAN rely largely on kidney biopsy, which provides histopathological confirmation but carries a risk of complications and does not reflect the systemic nature of the disease. Therefore, noninvasive biomarkers that facilitate early diagnosis and can be used to monitor disease progression, without the inherent risks associated with invasive procedures, are urgently needed. Recent studies have highlighted the potential of urinary metabolites and serum proteins as biomarkers of IgAN [5,6], but these findings have not been translated into clinical practice owing to their lack of specificity and sensitivity.

Considering these challenges, this study aimed to leverage Mendelian randomization (MR) as an analytical framework to elucidate the causal relationships between various biological exposures and IgAN outcomes [7]. MR uses genetic variants as instrumental variables (IVs) to circumvent confounding and rule out reverse causation, thereby providing stronger evidence for causal inference. By integrating proteomic and transcriptomic data through MR analysis, this study aimed to identify and validate novel plasma protein biomarkers associated with risk of IgAN development and progression.

To address these clinical needs, we used a multi-omics MR framework integrating proteome-wide association studies (PWAS) and single-cell transcriptome-wide association studies (scTWAS). By unmasking the causal network of circulating immune proteins, we aimed to identify reliable noninvasive biomarkers for IgAN and to provide new insights into the genetic mechanisms underlying the immune dysfunction of IgAN pathogenesis, laying the groundwork for the development of targeted, immune-modulating therapies.

## 2 Methods

### 2.1 Study design

The analysis pipeline was defined as follows (Fig. 1): (1) A two-sample MR method was used to select primary potential plasma protein targets for IgAN from a cohort of 35,559 Icelandic individuals through PWAS. Repeated validation was conducted on European cohorts of 54,219 individuals in the UK Biobank (UKB) and a cohort of 10,708 Fenland individuals. (2) Protein quantitative trait loci (pQTLs) of primary candidate plasma proteins were selected and colocalization analysis was performed with IgAN and the most statistically significant single-nucleotide polymorphisms (SNPs) (with the least *P*) to explore whether the SNP levels and IgAN were affected by the same variation within a certain range. (3) The expression of candidate plasma proteins in peripheral blood mononuclear cells (PBMCs) was verified using Gene Expression Omnibus (GEO, https://www.ncbi.nlm.nih.gov/gds/) datasets and a transcriptome-wide association study (TWAS) of candidate proteins was conducted with an expression quantitative trait locus (eQTL) to validate the association between candidate proteins and IgAN. (4) To verify the association between the expression of candidate proteins in specific cell subtypes and IgAN, a GEO single-cell transcriptome sequencing dataset was used to identify potential cell subtypes carrying the candidate proteins using single-cell specific eQTL (sceQTL) data from OneK1K (https://onek1k.org/). (5) Participation of the candidate protein targets in intercellular interactions in specific cell subtypes was verified through cell-communication analysis to reveal the cellular interactions of patients before and after tonsillectomy. (6) The effects of candidate protein targets were validated by MR replication analysis of common clinical manifestations of IgAN and kidney function. (7) Potential biological mechanisms for inferring protein targets were explored through protein-protein interaction (PPI) and Gene Ontology (GO) enrichment analysis. (8) Plasma levels of the target proteins in patients with IgAN and healthy controls were compared using enzyme-linked immunosorbent assays (ELISA).

**Fig. 1:**
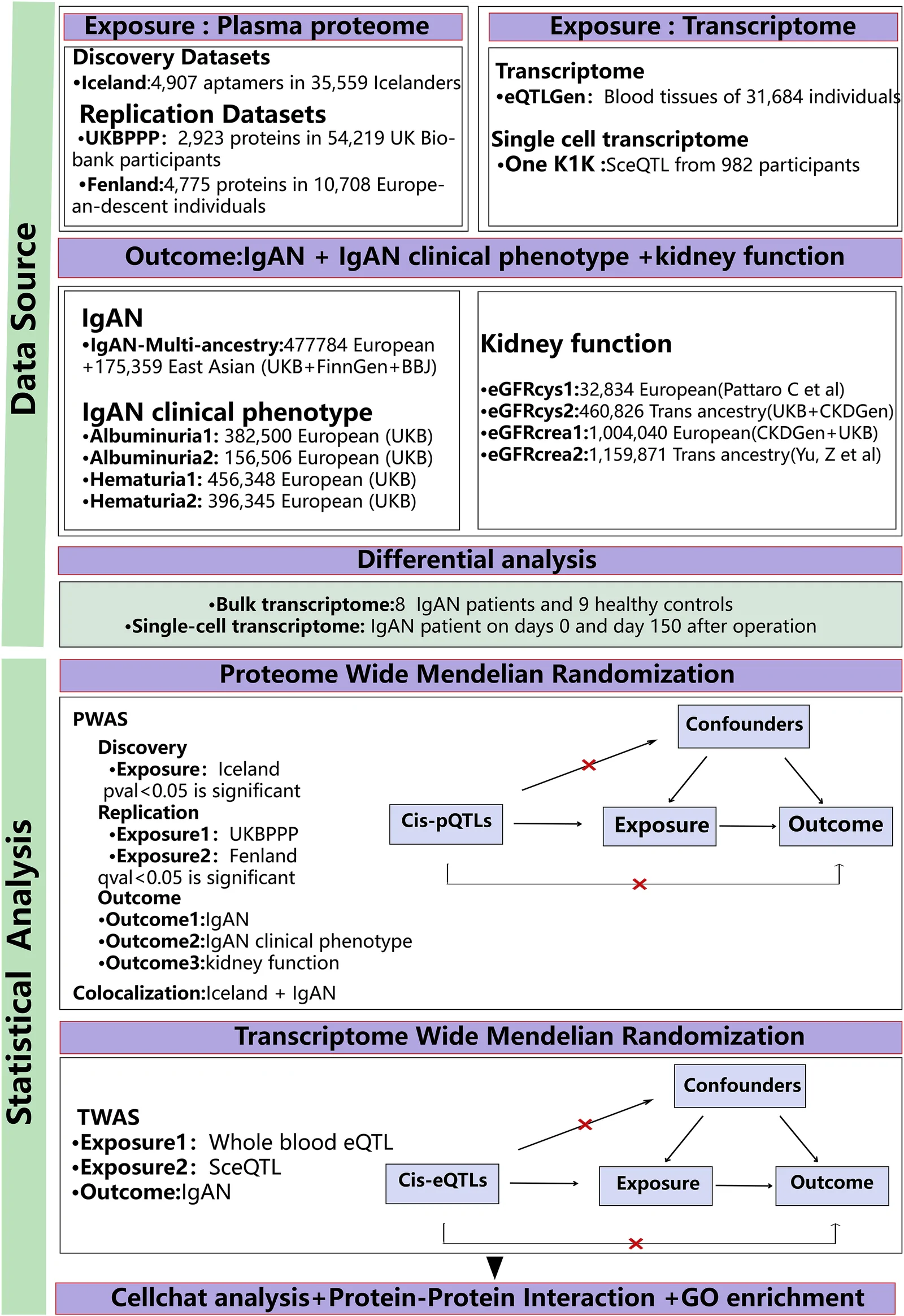
Flowchart of data sources and Mendelian randomization principles and methods.

### 2.2 Exposure data sources

The exposure data were derived from summary statistics of three large GWAS datasets: (1) Plasma protein genetic associations were obtained from deCODE genetics (Reykjavik, Iceland). A large-scale proteomic study was conducted of 40,004 Icelanders (52% from the Icelandic Cancer Project and 48% from various genetic programs at deCODE Genetics, Reykjavik) [8]. A total of 35,559 blood samples were selected to measure protein levels using SomaScan version 4 (SomaLogic, Boulder, CO, USA), which identified 28,191 genetic associations (*P* < 1.8 × 10^−9^). (2) A total of 10,674 genetic associations (8328 sentinel signals and 2346 secondary signals, *P* < 1.004 × 10^−11^) for 3892 plasma proteins identified in 10,708 participants in the Fenland cohort (Cambridgeshire, UK) [9]. (3) A total of 23,588 sentinel genetic associations (*P* < 1.7 × 10^−11^) for 2923 plasma proteins were identified in 54,219 participants from the UK Biobank Pharma Proteomics Project (UKB-PPP) using the Olink platform [10]. Cis-pQTLs were defined as genetic variants within 1 Mb upstream or downstream of the transcription start site of the protein-encoding gene, whereas trans-pQTLs were defined as variants that influenced protein levels but exceeded the cis-regulatory region.

### 2.3 Outcome data sources

The multi-ancestry IgAN dataset combined the GWAS data of individuals with European ancestry (15,587 cases and 462,197 controls) from the UK Biobank (UKB) and FinnGen cohorts, and individuals with East Asian ancestry (71 cases and 175,288 controls) from the Japan Biobank [11]. The original study used a generalized linear mixture model implemented in SAIGE (version 0.37) to perform GWAS on IgAN, and individuals with mutations within the major histocompatibility complex (MHC) region were excluded.

Data on albuminuria were obtained from two GWAS datasets: (1) Quantitative analysis of urine albumin in samples from 382,500 unrelated European individuals from UKB [12]. (2) Microalbuminuria measured using immunoturbidimetric analysis of samples from 156,506 European individuals, obtained from UKB (data field:30500).

Data on hematuria were obtained from two large GWAS datasets: (1) GWAS data of 456,348 individuals of European ancestry (15,001 cases and 441,347 controls) from UKB, simulated and corrected using the fastGWA method based on a generalized linear mixed model (GLMM) [13]. (2) UKB GWAS data on hematuria obtained from 396,345 samples of British nationals of European descent (16,409 cases and 379,936 controls) corrected for imbalanced case-control ratios using generalized mixed models (SAIGE) [14].

Data on kidney function were obtained from two datasets of the glomerular filtration rate (GFR) estimated from both serum creatinine (eGFRcrea) and serum cystatin C (eGFRcys) levels. First, two eGFRcys GWAS datasets were included: (1) GWAS data on eGFRcys from 16 cohorts of 32,834 individuals of European ancestry [15]. (2) GWAS data on eGFRcys of 460,826 individuals with European or unknown ancestry from UKB and CKDGen [16]. Second, we also derived two eGFRcrea GWAS datasets: (1) GWAS data of eGFRcrea, of 1,004,040 individuals with European ancestry were sourced from the CKDGen consortium (n = 436,561, European statistics, application number 20272) and UKB (n = 765,348, mostly Europeans). The EGFRcrea data were winsorized at 15 and 200 mL/min/1.73 m^2^ [16]. (2) Multi-ancestry GWAS data of eGFRcrea of 949,116 individuals of European descent, 166,997 individuals of East Asian descent, 21,338 individuals of South Asian descent, 17,459 African-American individuals and 4961 Hispanic-American individuals were obtained from a follow-up survey of the Atherosclerosis Risk in Communities (ARIC) research project, conducted every 3 years in four communities in the United States [17].

### 2.4 Transcriptome data sources

For differential expression analysis, transcriptome expression data on mRNA expression profiles in the PBMCs of 8 patients with IgAN and 9 healthy controls from a study conducted in Italy were source from GEO (GEO series: GSE58539) [18].

To verify the association between candidate proteins and IgAN at a transcriptome level, another TWAS analysis was conducted using data on whole blood samples integrated from 37 datasets in eQTLGen Consortium, which included a total of 31,684 individual samples [19].

To investigate the potential effect of candidate proteins in various cell subtypes in peripheral blood, we used PBMC data from Sun Yat-sen University in China, which included single-cell transcriptome sequencing data on PBMCs from patients with IgAN before tonsillectomy (Day 0) and 150 days after tonsillectomy (Day 150) (GEO series: GSE189481) [20]. All comparisons in this study were made within the same patients between these two timepoints; no independent healthy control group was included in this dataset.

To investigate the effect of candidate protein expression in specific cell subtypes on IgAN, single-cell specific eQTL (sceQTL) data, consisting of 1.7 million PBMCs from 982 blood donors, were obtained from One K1K for use as IVs for scTWAS [21].

### 2.5 Two-sample Mendelian randomization

We used a two-sample MR approach to identify potential plasma protein targets for IgAN [22]. The analysis was conducted based on the three core assumptions of MR (Fig. 1): (1) The IVs must be strongly associated with the exposure; (2) The IVs must influence the outcome exclusively through the exposure; and (3) The IVs must be independent of any confounding factors which may also influence the outcome. To ensure that these assumptions were met, we implemented the following measures: We restricted our IVs to cis-pQTLs with *P* < 5 × 10^−8^ and calculated the *F*-statistic for each cis-pQTL to evaluate its strength. For a single pQTL, the *F*-statistic was approximated as: $F=\frac{\beta^{2}}{{SE}^{2\;}}$ [23], where *β* is the effect of the pQTL on protein levels, and SE is the standard error of the association [22]. We filtered out IVs with *F*-statistic values < 10 and used the ld_clump function from the ‘ieugwasr’ package to filter pQTLs in the European reference genome with parameters clump_kb = 10000 and clump_r2 = 0.001, to control for the influence of linkage disequilibrium (LD) [19].

The results of this study were reported according to the STROBE-MR guidelines [22]. The completed STROBE-MR checklist is provided as Supplementary File 3. For proteins with a single cis-pQTL, the Wald ratio method was used to estimate the causal effect size (β) and 95% corresponding confidence interval (CI) [24], whereas for proteins with multiple cis-pQTLs, estimates were obtained using the inverse-variance weighted (IVW) method [25]. In the two-sample MR analysis, the Icelandic cohort was used as the discovery dataset. In this exploratory phase, we attempted to discover as many potential positive results as possible, so we did not perform P-value correction, and P-values < 0.05 were considered significant [26]. Repeated MR analysis of the primary candidate plasma proteins were subsequently performed using the Fenland and UKB-PPP cohorts as replication datasets and the results were corrected for the False Discovery Rate (FDR). Corrected q-values < 0.05 were considered statistically significant [26].

This study used the Cochran *Q*-test to evaluate heterogeneity. If the *P*-value was > 0.05, indicating a lack of significant heterogeneity, the fixed-effects IVW method was used. However, if the P-value was < 0.05, indicating substantial heterogeneity, then the random-effects IVW method was used [27].

The MR-Egger method was used to test for directional (horizontal) pleiotropy [28]. We tested that the MR-Egger regression intercept had a threshold *P*-value > 0.05 to rule out the presence of significant horizontal pleiotropy. A nonsignificant intercept term indicates that the horizontal pleiotropy of the IVs is within a controllable range.

The MR and sensitivity analyses were performed using ‘TwoSampleMR’ R package (version 0.6.8) [19].

### 2.6 Transcriptome analysis

We used the ‘limma’ package in R to perform differential expression analysis with the transcriptome sequencing data [29]. Logarithmic conversion and standardization of the raw data were performed to control for heteroscedasticity. The eBayes function was used to build a linear model fitting standardized data and to conduct empirical Bayesian adjustment of the standard error. Significance was determined based on *P* < 0.05.

We performed a TWAS using cis-eQTLs from eQTLGen as instruments for gene expression (exposure) and IgAN as the outcome.

### 2.7 Colocalization

Bayesian colocalization is a tool that uses SNPs as IVs to evaluate whether two traits share the same causal variation. It makes the following five hypotheses: (1) H0: Not associated with any trait; (2) H1: Only associated with trait A; (3) H2: Only associated with trait B; (4) H3: Associated with both traits A and B within the region but not sharing the same variation; and (5) H4: Both traits A and trait B are associated within the region and share the same variation [30].

We performed Bayesian colocalization of the primary candidate plasma protein and IgAN within 1 Mb upstream or downstream of the leading SNP of the primary candidate plasma protein to evaluate the probability of sharing the same causal variation. We used the ‘coloc’ R package with default parameters to perform colocalization [30]. To ensure that the number of SNPs within the selected range did not exceed 10,000, the colocalization range of plasma proteins with more than 10,000 SNPs shared in the IgAN outcome within the selected range was adjusted to 350 kb upstream and downstream of the leading SNP. Owing to the limited validation effectiveness of colocalization, we focused on plasma proteins with *PP.H4* >0.75 or *PP.H3 + PP.H4* >0.8 [31].

### 2.8 Single-cell transcriptome analysis

We used the ‘Seurat’ R package to establish Seurat objects based on single-cell transcriptome sequencing data with the parameters set at min.cells = 5, min.features = 300 [32]. To ensure a homogeneous cell population with uniform sequencing depth for downstream analysis, a stringent quality control strategy was implemented. The specific criteria were as follows: proportion of hemoglobin genes (pHB) < 0.05, nFeature_RNA > 1000, and a strictly confined nCount_RNA range (9975 to 9999) to minimize the potential for bias originating from variable sequencing depths. Principal component analysis (PCA) was performed to reduce the dimensionality with the number of principal components (nPCs) = 50. T-Distributed Stochastic Neighbor Embedding (t-SNE) was applied with nPCs = 18 to visualize the cell populations. The cells were clustered using the Louvain algorithm at a resolution of 0.5.

For IVs in the scTWAS, we performed LD clumping with a threshold of r2 < 0.01, using sceQTLs that were associated with gene expression levels with *P <* 0.05 and *F >* 10 [33]. The causal effects of candidate protein-coding gene expression within specific cell subtypes on the IgAN outcome were evaluated using the IVW, Simple mode, Weighted mode, Weighted median, and MR-PRESSO methods. *P*-values < 0.05 were considered statistically significant, and potential pleiotropy was assessed using the MR-PRESSO framework [34].

The ‘CellChat’ R package with default parameters was used to analyze intercellular signal transduction [35]. In addition, the human reference ligand table provided by CellChat, supplemented receptor-ligand pairs provided by the UniProt database were used in this study (Supplementary File 2, Table S1) [36].

### 2.9 PPI and GO enrichment analysis

For plasma proteins that could be validated as significantly associated with IgAN in two or more datasets, as well as plasma proteins with positive colocalization results, including some that were significantly associated with IgAN only in the Icelandic dataset, we constructed PPI networks using STRING and performed GO enrichment analysis on proteins that could form interactions with other proteins in the network [37].

### 2.10 Measurement of plasma protein concentrations

Plasma samples were collected from 28 patients with IgAN and 14 healthy controls at the First Affiliated Hospital of Gannan Medical University, China. The healthy controls were individuals attending the health examination centre of the same hospital; all had normal renal function and a normal urinalysis, being negative for both occult blood and protein. Demographic and clinical characteristics of both groups are provided in Supplementary File 2, Table S2A and S2B. The study was approved by the Ethics Review Committee of the First Affiliated Hospital of Gannan Medical University (approval number LLSC2023185). Written informed consent was obtained from all participants, or from a parent or legal guardian for participants under 18 years of age, and the study was conducted in accordance with the Declaration of Helsinki. Plasma AIF1 concentrations were determined by a commercial sandwich ELISA kit (Cat. No. HM12489; Bioswamp, Wuhan, China) according to the manufacturer's instructions, with absorbance read at 450 nm. Assays were performed by Bioswamp (Wuhan, China). The Mann–Whitney *U* test was used to compare the plasma AIF1 levels between patients with IgAN and healthy controls, and P-values < 0.05 were considered statistically significant.

## 3 Results

### 3.1 Association between plasma protein levels and IgAN

In the Icelandic discovery cohort, 92 of the 1763 plasma proteins with valid instruments after LD clumping were significantly associated with IgAN (Fig. 2A). Detailed data on these associations in the discovery datasets and the results of the sensitivity analysis are shown in Supplementary File 2, Table S3.

**Fig. 2:**
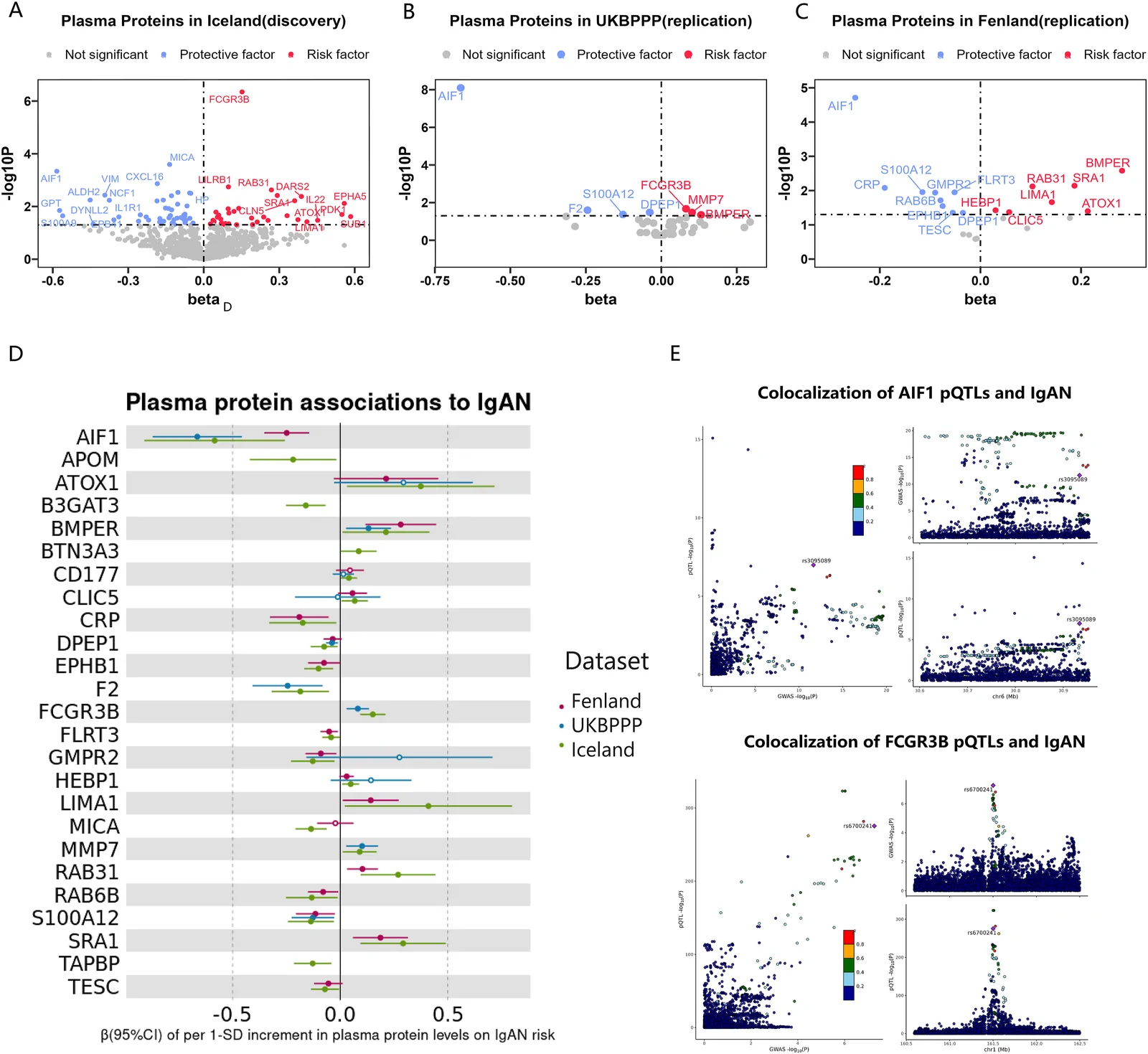
**Associations between plasma protein levels and IgA nephropathy risk across three cohorts, and genomic colocalization analysis.** A) Volcano plot of plasma protein MR results in the Icelandic (discovery) cohort (beta vs. −log10P; blue = protective factor, red = risk factor). B) Same analysis in the UKB-PPP (replication) cohort. C) Same analysis in the Fenland (replication) cohort. D) Integrated forest plot of plasma protein–IgAN associations across the three cohorts, with horizontal bars representing the 95% CI. E) Regional colocalization plots (LocusCompare) for AIF1 (top) and FCGR3B (bottom) pQTLs and IgAN GWAS signals, colored by linkage disequilibrium (r^2^) with the lead variant.

The associations between the 92 plasma proteins and IgAN obtained from the discovery dataset were validated in the two duplication datasets. After FDR correction, 7 plasma proteins in the UKB-PPP cohort and 16 plasma proteins in the Fenland cohort were significantly associated with IgAN (Fig. 2B and C). Detailed data of these associations in the duplication datasets and the results of the sensitivity analysis are shown in Supplementary File 2, Table S4. Through this rescreening, 19 unique proteins were identified as candidate targets.

After Bayesian colocalization analysis of 92 primary plasma proteins, 8 were found to positively colocalize with IgAN (Fig. 2E,Table 1, Supplementary File 1, Fig. S1 and Supplementary File 2, Table S5). FCGR3B (*PP.H4* = 93.11%), B3GAT3 (*PP.H3* = 6.36%, *PP.H4* = 75.15%), and MICA (*PP.H4* = 98.75%) were highly colocalized with IgAN (*PP.H4* > 75%). In addition, AIF1 (*PP.H3* ≈ 100%), APOM (*PP.H3* ≈ 100%), transporter associated with antigen processing (TAP)-binding protein (TAPBP) (*PP.H3* ≈ 100%), BTN3A3 (*PP.H3* = 81.63%, *PP.H4* = 18.37%), CD177 (*PP.H3* = 78.92%, *PP.H4 =* 1.19%) also satisfied the criteria for positive colocalization with IgAN (*PP.H3 + PP.H4* > 80%), although the lower PP.H4 meant that the colocalization may not be caused by the same variant, these plasma protein levels were closely related to IgAN risk within the prespecified genome range.

**Table 1:** Bayesian colocalization results.

exposure	nSNPs	PP.H0	PP.H1	PP.H2	PP.H3	PP.H4
AIF1	2554	0.000	0.000	0.000	1.000	0.000
FCGR3B	4831	2.518e-319	0.001	1.321e-317	0.068	0.931
MICA	2526	0.000	0.000	0.000	0.013	0.987
APOM	2716	0.000	0.000	0.000	1.000	0.000
TAPBP	7959	0.000	0.000	0.000	1.000	0.000
BTN3A3	5250	0.000	0.000	9.687e-318	0.816	0.184
B3GAT3	4171	0.000	0.185	0.000	0.064	0.751
CD177	5592	3.079e-316	0.199	1.222e-315	0.789	0.012

Although MICA, B3GAT3, APOM, TAPBP, CD177, and BTN3A3 were validated only in the MR of the Icelandic data, their positive colocalization indicated their potential as IgAN targets (Fig. 2E and Supplementary File 1, Fig. S1). As a negative colocalization result does not necessarily invalidate the MR results, an additional 6 proteins were included along with the 19 previously identified proteins to explore candidate targets.

Among these plasma proteins, AIF1, BMPER, DPEP1, and S100A12 were validated as significant in all three cohorts. Their *β* values (95% CIs) were −0.584 (−0.910 to −0.257), 0.213 (0.010 to 0.415), −0.075 (−0.138 to −0.012) and −0.136 (−0.243 to −0.030) in the discovery dataset. Fifteen proteins, including FCGR3B, LIMA1, MMP7, RAB31, RAB6B, and SRA1 were validated as significant in two of the three cohorts. Their *β* values (95% CIs) were 0.151 (0.092 to 0.210) for FCGR3B, 0.410 (0.021 to 0.799) for LIMA1, 0.090 (0.011 to 0.169) for MMP7, 0.269 (0.095 to 0.443) for RAB31, −0.132 (−0.252 to −0.012) for RAB6B, and 0.292 (0.094 to 0.491) for SRA1. In addition, in the discovery dataset six other plasma proteins, MICA, APOM, TAPBP, CD177, B3GAT3, and BTN3A3, colocalized with IgAN. Their *β* values (95% CIs) were −0.14 (−0.21 to −0.06) for MICA, −0.22 (−0.42 to −0.02) for APOM, −0.13 (−0.22 to −0.04) for TAPBP, 0.04 (0.00 to 0.08) for CD177, −0.16 (−0.25 to −0.07) for B3GAT3, and 0.09 (0.00 to 0.17) for BTN3A3 (Supplementary File 2, Table S3). Fig. 2D shows a summary of the associations between these 25 plasma proteins and IgAN in the three cohorts. In this study, the Cochran Q-test indicated no significant heterogeneity, and the fixed-effects IVW model was therefore used. All 25 proteins showed nominally significant associations with IgAN in at least one cohort, and were carried forward as candidate targets for downstream validation.

### 3.2 Associations of the protein-coding gene expression level with IgAN

*AIF1* expression was significantly downregulated in PBMCs of patients with IgAN, (logFC = −0.156, *P* = 0.036) (Fig. 3A and Supplementary File 2, Table S6). In addition, *CLIC5* (logFC = −0.086, *P* = 0.045, Italy), and B3GAT3 (logFC = 0.174, *P =* 0.050, Italy) expression levels were also significantly associated with IgAN.

**Fig. 3:**
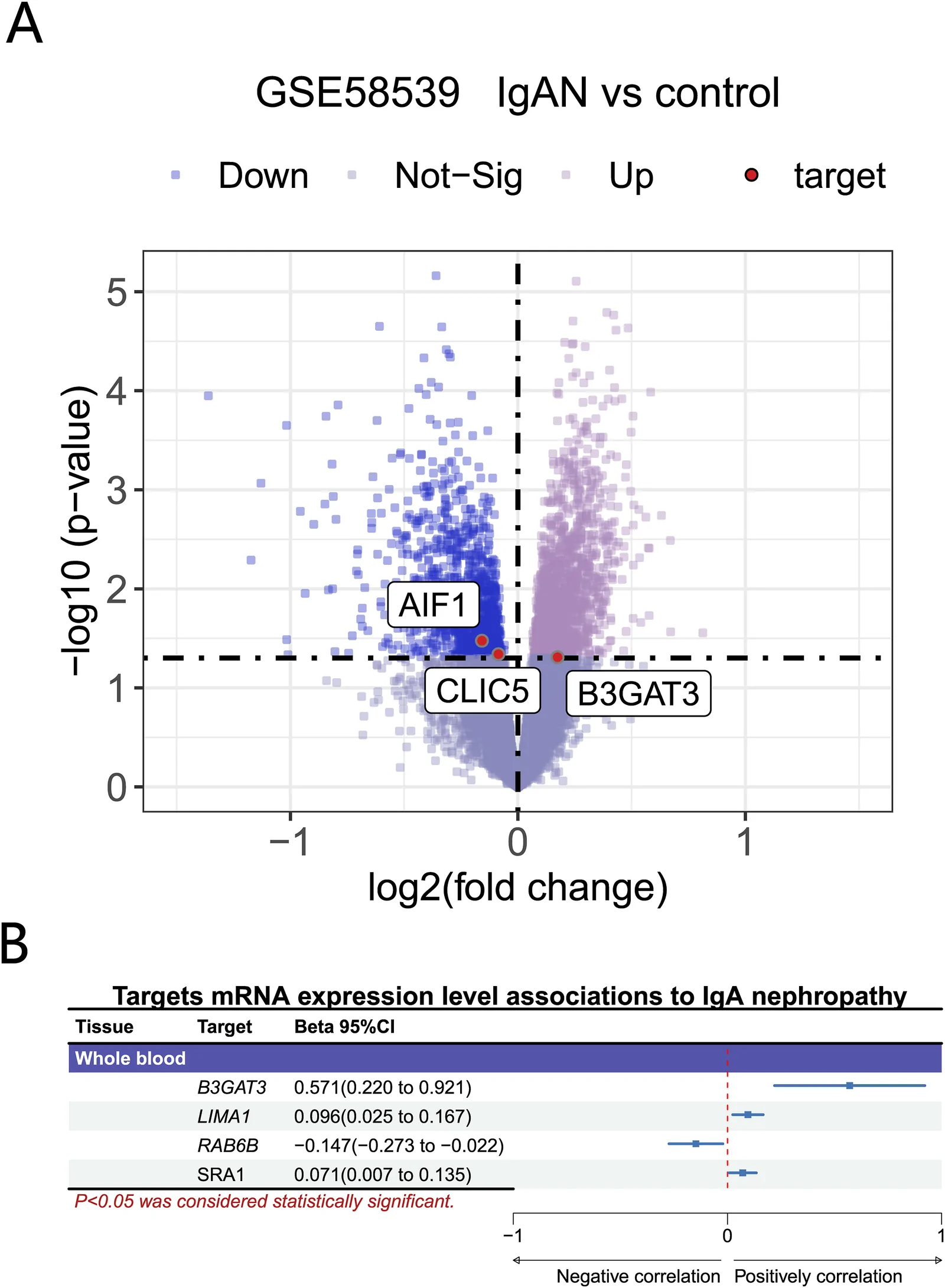
**Associations between candidate gene expression levels and IgA nephropathy risk at the transcriptome level**. A) Volcano plot of differences in peripheral blood samples between patients with IgA nephropathy (IgAN) and healthy controls, with purple red representing upregulation and blue representing downregulation. B) Forest plot of Mendelian randomization (MR) analysis of the association between mRNA expression levels of candidate genes and IgAN risk.

The TWAS results of candidate proteins using transcriptome data from eQTLGen showed that the direction and magnitude of the association of *LIMA1*, *SRA1*, and *RAB6B* with IgAN risk at the transcriptome level were consistent with those at the proteome level (Fig. 3B and Supplementary File 2, Table S7), confirming that they were associated with IgAN. The *β* values (95% CIs) of the expression of the *LIMA1* and *SRA1* on IgAN were 0.096 (0.025 to 0.167) and 0.071 (0.007 to 0.135), respectively, indicating that they were associated with an increased risk of IgAN. The *β* value (95% CI) of the expression level of *RAB6B* on IgAN was −0.147 (−0.273 to −0.022), indicating a protective effect on IgAN. However, for B3GAT3 the TWAS results were inconsistent with the PWAS results, making its effect unclear. The β value (95% CI) of B3GAT3 expression in whole blood of patients with IgAN was 0.571 (0.220 to 0.921), showing a positive association, which was opposite to the corresponding PWAS result.

### 3.3 Single-cell transcriptome analysis to identify PBMC cell types in which candidate proteins play a role

After clustering, the Seurat object was divided into eight cell subtypes, namely: monocytes, CD4^+^ T cells, CD8^+^ T cells, B cells, effector memory T (Tem) cells, natural killer (NK) cells, macrophages, and dendritic cells (Fig. 4A).

**Fig. 4:**
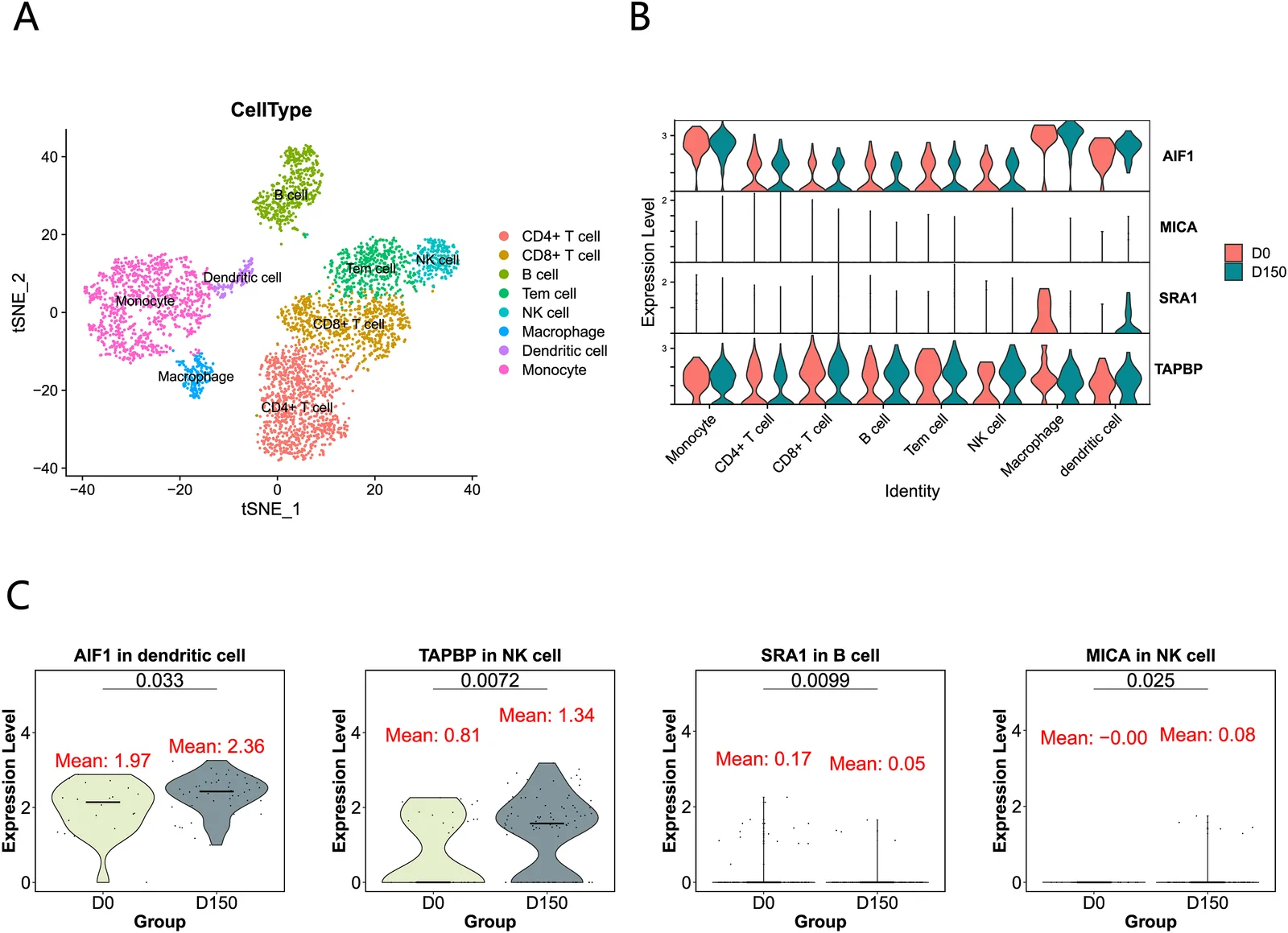
**Single-cell transcriptomic analysis of candidate gene expression across PBMC subpopulations before and after tonsillectomy**. A) t-distributed stochastic neighbor embedding (t-SNE) plot of peripheral blood mononuclear cell (PBMC) single-cell subsets in patients with IgA nephropathy (IgAN). B) Violin plot showing the expression levels of AIF1, MICA, SRA1, and TAPBP across PBMC cell subpopulations. C) Violin plots comparing AIF1 expression in dendritic cells, TAPBP expression in NK cells, SRA1 expression in B cells, and MICA expression in NK cells before (Day 0) and after (Day 150) tonsillectomy.

The differential analysis of the genes encoding the candidate proteins expressed in various cell subtypes of patients with IgAN before and 150 days after tonsillectomy showed that the preoperative and postoperative expression levels did not differ significantly in most cell subtypes. However, the expression levels of *AIF1*, *MICA*, *TAPBP*, and *SRA1* differed significantly in specific cell subtypes (Fig. 4B and C).

In dendritic cells, the *AIF1* expression level in patients with IgAN was significantly higher than the preoperative level at 150 days postoperatively (*P* = 0.033). In B cells, the *SRA1* expression level in patients with IgAN was significantly lower than the preoperative level at 150 days postoperatively (*P* = 0.010). In NK cells, the *MICA* and *TAPBP* expression levels in patients with IgAN were significantly higher than the preoperative levels at 150 days postoperatively (*P* = 0.025 and *P* = 0.007, respectively).

The expression levels of *AIF1* in dendritic cells, and *MICA* and *TAPBP* in NK cells, were negatively correlated with IgAN, whereas the expression level of *SRA1* in B cells was positively correlated with IgAN, consistent with the PWAS results, indicating that these cell types may be key sites for target proteins.

### 3.4 Potential pathways of AIF1 action in dendritic cells

The scTWAS results of the associations of proteins with significant expression differences between specific cell subtypes and IgAN showed that the *AIF1* expression levels in dendritic cells estimated were consistently negatively correlated with IgAN using the five MR methods (Fig. 5A). The *β* (95% CI) were: −0.049 (−0.105 to 0.006) for Weighted median, −0.047 (−0.090 to −0.005) for IVW, −0.050 (−0.125 to 0.024) for Simple mode, −0.050 (−0.123 to 0.023) for Weighted mode, and −0.047 (−0.054 to −0.041) for MR-PRESSO (Supplementary File 2, Table S8). The *P*-values for the IVW method and MR-PRESSO were 0.02875 and 6.28E−7, respectively. The Cochran Q-test did not reveal any significant heterogeneity, and in the MR-PRESSO test did not reveal any obvious outliers or pleiotropy.

**Fig. 5:**
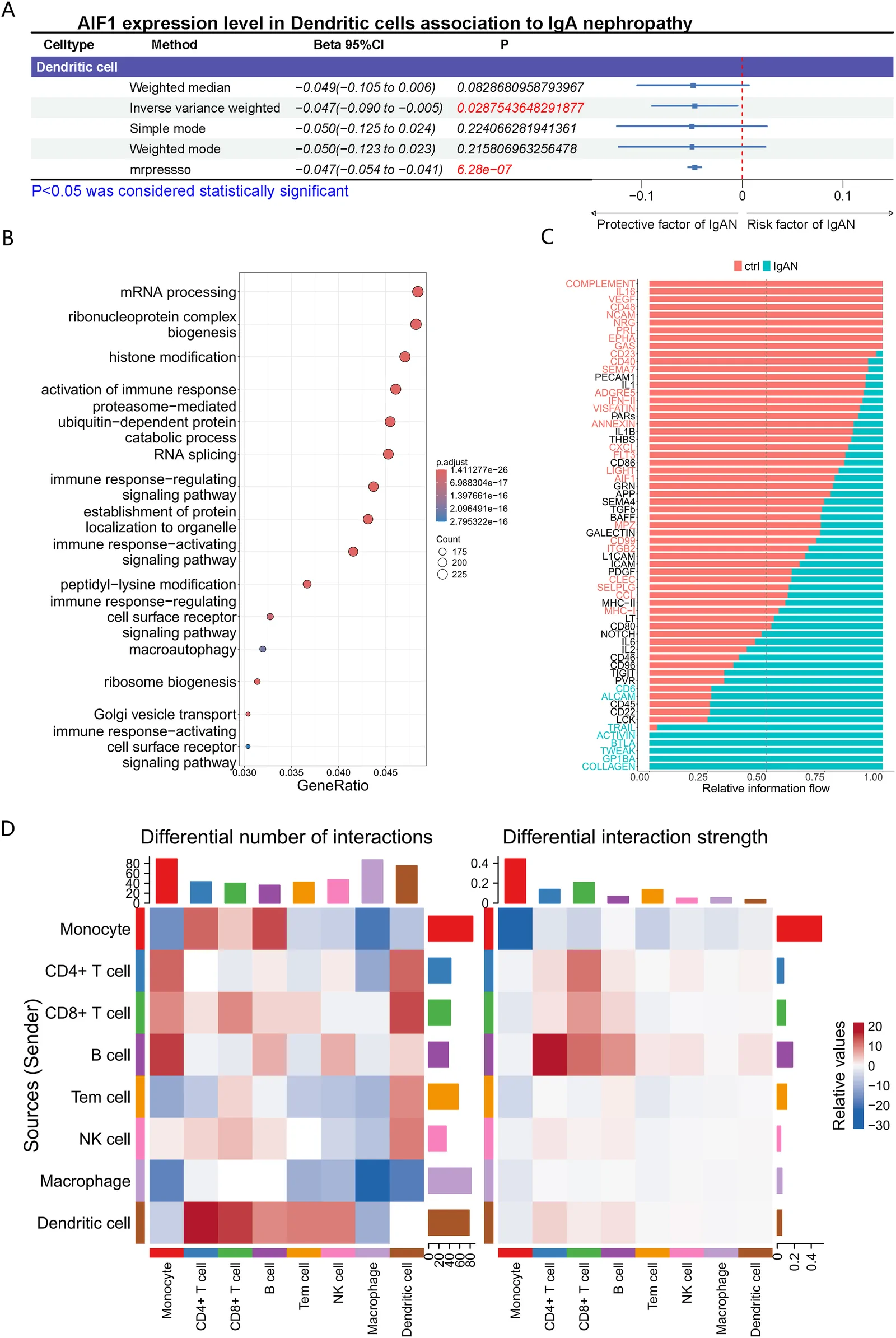
**AIF1-mediated dendritic cell signaling pathways in IgA nephropathy**. A) Forest plot of the association between specific AIF1 expression levels in dendritic cells and risk of IgA nephropathy (IgAN), calculated using five Mendelian randomization (MR) algorithms. B) Bubble plot of top 20 BP results of GO enrichment of differentially expressed genes upregulated after surgery. C) Differences in the intensity of cell ligand receptor enrichment pathways before and after surgery. Ctrl denotes Day 150 (post-tonsillectomy) and IgAN denotes Day 0 (pre-tonsillectomy) in the same patients. D) Heatmap of changes in signal number and strength output and reception of various cell subpopulations after surgery compared with those before surgery.

The GO terms for the top 20 differentially expressed genes (DEGs) between dendritic cells at Day 0 and Day 150 are shown in Fig. 5B. These genes were mainly enriched in mRNA production, ribosome complex production, and histone modification, and were also highly enriched in immune-related pathways such as the immune activation response, immune response regulation signaling pathway, immune response activation signaling pathway, and cell surface receptor signaling pathway that regulate immune response. The full list of GO enrichment terms is provided in Supplementary File 2, Table S9.

The summary of cellular communication pathways showed that the AIF1-related pathway intensity in dendritic cells was higher at Day 150 than at Day 0. In addition, 43 pathways, including key immune-related pathways, such as complement, IL16, CD48, CD23, and CD40, had higher intensity at Day 150 than at Day 0 (Fig. 5C).

After surgery AIF1 was significantly upregulated and the expression of receptor pairs responsible for transmitting signals from dendritic cells to CD4^+^ T cells, CD8^+^ T cells, macrophages, and monocytes changed significantly (Fig. S2), compared with the signals before surgery (Fig. S3). The differences in AIF1 receptor pairs indicate the important role of AIF1 in dendritic cell signaling to other cells.

Compared with the period of illness, patients with IgAN showed significant changes in unidirectional signaling between eight cell subtypes in the peripheral blood after tonsillectomy (Fig. 5D). With higher AIF1 levels, the number of signals transmitted from dendritic cells to CD4^+^ T cells, CD8^+^ T cells, B cells, Tem cells, and NK cells increased significantly after surgery, whereas the number of signals transmitted to macrophages and monocytes significantly decreased. In terms of signal transmission intensity, overall, little difference was observed between Day 0 and Day 150, but Day 150 showed slightly higher signal transmission from dendritic cells to CD4^+^ T cells, CD8^+^ T cells, and B cells. In summary, AIF1 expressed in dendritic cells is involved in the regulation of immune processes.

### 3.5 Repeated MR analysis to validate of the association between candidate plasma proteins and common clinical manifestations of IgAN and indicators of kidney function

The MR validation of indicators of kidney function using candidate plasma protein pQTLs from the Icelandic cohort revealed that 9 plasma proteins were significantly associated with eGFRcrea (Fig. 6A and Supplementary File 2, Table S10) and 7 plasma proteins were significantly associated with eGFRcys (Fig. 6B and Supplementary File 2, Table S11).

**Fig. 6:**
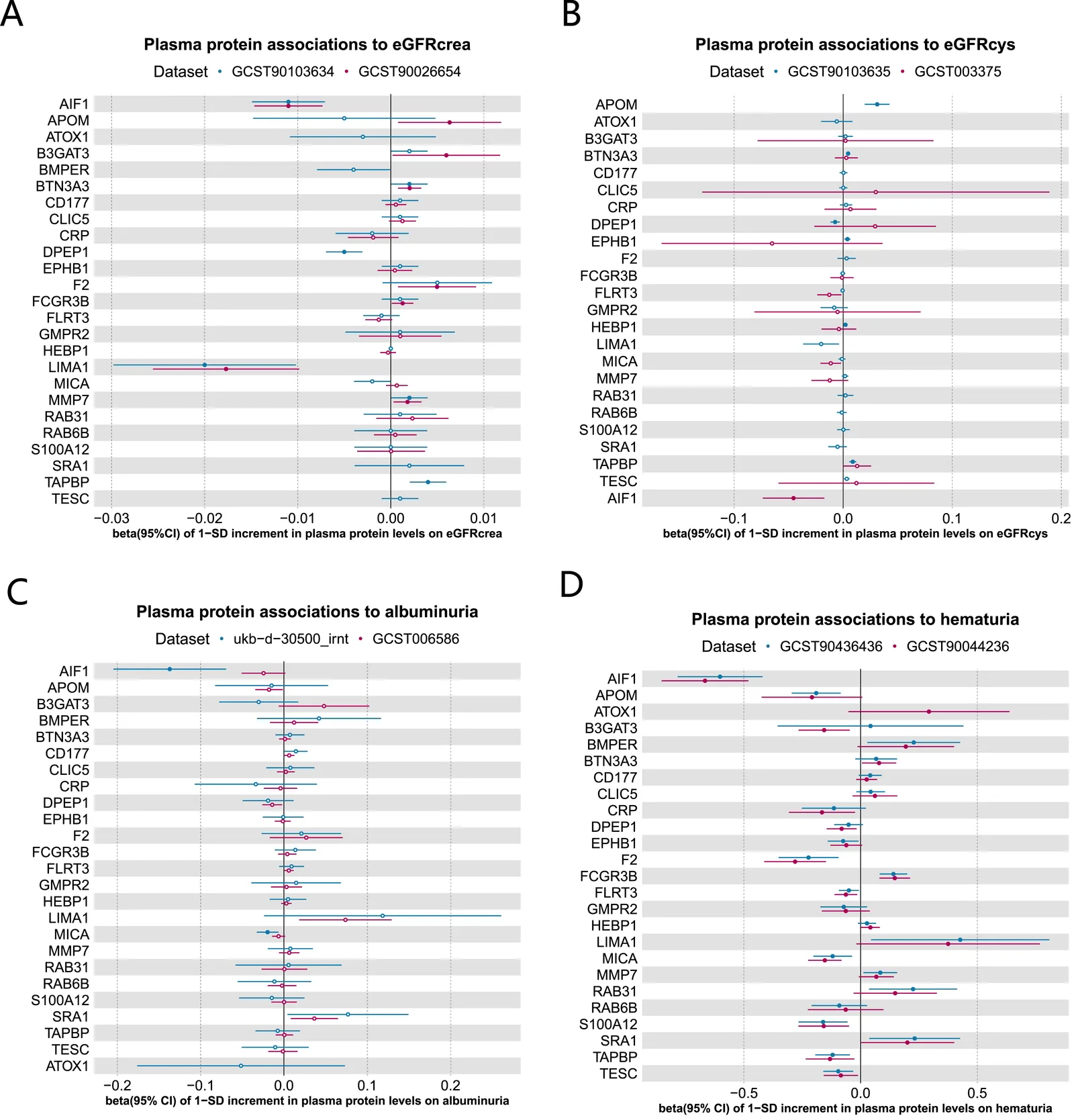
**MR analysis forest plot of the association between plasma protein levels and common clinical manifestations of IgAN**. A) Serum creatinine (eGFRcrea). B) Serum cystatin C (eGFRcys). C) albuminuria. D) hematuria.

For eGFRcrea, AIF1, LIMA1, and DPEP1 showed significant negative correlations, with AIF1 (β = −0.011, 95% CI: −0.015 to −0.007) and LIMA1 being validated in two outcome datasets, whereas BTN3A3, MMP7, and TAPBP were positively correlated with eGFRcrea in both datasets.

For eGFRcys, AIF1 and DPEP1 levels were significantly negatively correlated with eGFRcys and were validated as significant only in the dataset ebi-a-GCST003375. The *β* values (95% CIs) were −0.046 (−0.074 to −0.017) for AIF1, and −0.007 (−0.012 to −0.003) for DPEP1, respectively (ebi-a-GCST003375). The levels of BTN3A3, EPHB1, HEBP1, TAPBP, and APOM were significantly positively correlated with eGFRcys and were validated as significant only in outcome data ebi-a-GCST90103635. Their *β* values (95% CIs) were 0.005 (0.003 to 0.006) for BTN3A3, 0.004 (0.001 to 0.007) for EPHB1, 0.002 (0.000 to 0.004) for HEBP1, 0.009 (0.006 to 0.012) for TAPBP, and 0.031 (0.020 to 0.043) for APOM.

MR validation of the common clinical manifestations of IgAN showed that 2 plasma protein levels were significantly correlated with albuminuria (Fig. 6C and Supplementary File 2, Table S12), and 20 plasma protein levels were significantly correlated with the risk of hematuria (Fig. 6D and Supplementary File 2, Table S13).

For albuminuria, AIF1 and MICA levels were significantly negatively correlated with levels of microalbuminuria, and these results were validated as significant only in the outcome dataset ebi-a-GCST006586. The *β* values (95% CIs) were −0.137 (−0.205 to −0.069) for AIF1 and −0.020 (−0.033 to −0.007) for MICA.

Twelve plasma proteins showed significant negative correlations with hematuria. Notably, seven of these targets, including the core molecule AIF1, were validated in two independent outcome datasets (ebi-a-GCST90044236 and ebi-a-GCST90436436). AIF1 exhibited the strongest protective effect against hematuria (*β* = −0.667, 95% CI: −0.853 to −0.481 in ebi-a-GCST90044236). Conversely, eight proteins were positively correlated with the risk of hematuria, with FCGR3B being the only risk factor validated in both datasets (*β =* 0.146, 95% CI: 0.081 to 0.212 in ebi-a-GCST90044236). The detailed results for the other single- and double-validated targets are summarized in the supplementary tables (Supplementary File 2, Table S13).

AIF1 was significantly negatively correlated with both albuminuria and hematuria, which suggests that it has a protective effect against IgAN.

### 3.6 PPI network and Gene Ontology enrichment of candidate proteins

The interaction analysis revealed that AIF1, FCGR3B, CD177, S100A12, CRP, F2, and APOM can interact, and that BTN3A3 and MICA can interact (Fig. 7A). The GO enrichment analysis of the biological pathways of 25 plasma proteins involved in PPIs revealed that these proteins were mainly involved in defense responses against bacteria, leukocyte-mediated immunity, positive regulation of reaction processes, oxygen metabolism processes, cell killing, positive regulation of superoxide anion production, regulation of superoxide anion generation, regulation of superoxide anion generation metabolism processes, and neutrophil-mediated immunity (Fig. 7B). The GO enrichment results of the proteins involved are shown in Supplementary File 2, Table S14.

**Fig. 7:**
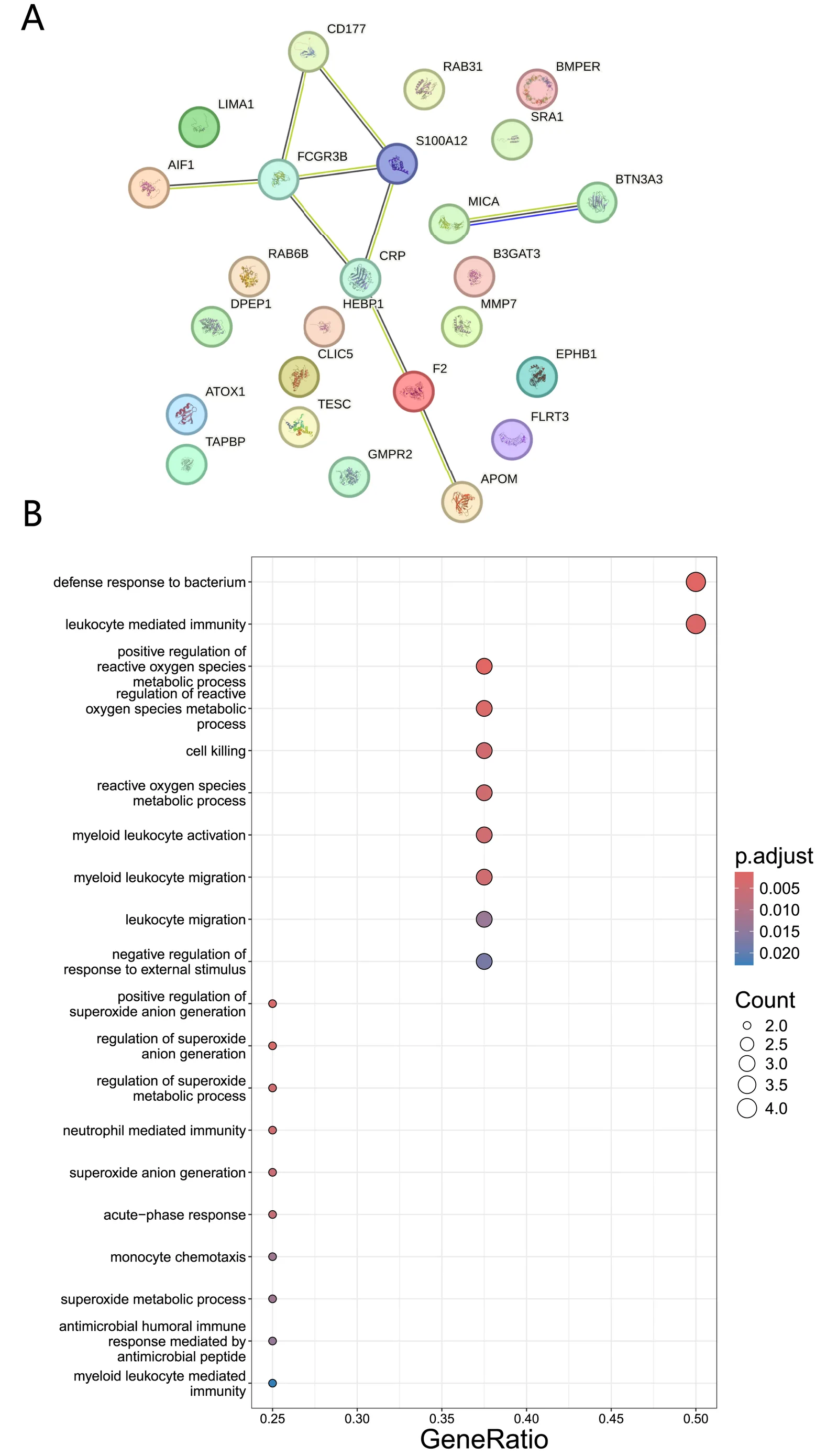
**Protein-protein interaction network and functional enrichment analysis of candidate plasma proteins**. A) Protein-protein interaction (PPI) interaction network diagram between candidate genes. B) Bubble plot of biological processes (BP) obtained from Gene Ontology (GO) enrichment analysis of protein targets forming local modules.

### 3.7 Plasma AIF1 levels in patients with IgAN and healthy controls

A comparison of the plasma AIF1 levels of patients with IgAN (n = 28) and healthy controls (n = 14), measured by ELISA, is shown in Fig. 8. The IgAN group had a median age of 33 years (range 15–58) and comprised 39.3% men; the healthy control group had a median age of 32 years (range 19–56) and comprised 42.9% men. The two groups were not formally matched, but did not differ significantly in age (P = 0.894, Mann–Whitney *U* test) or in sex distribution (P = 1.000, Fisher's exact test). The median plasma AIF1 concentration was 438.46 pg/mL in healthy controls and 325.07 pg/mL in patients with IgAN (P = 0.044, Mann–Whitney *U* test), indicating significant downregulation of AIF1 in patients with IgAN compared with healthy controls. The plasma AIF1 protein results for each sample are shown in Supplementary File 2, Table S15.

**Fig. 8:**
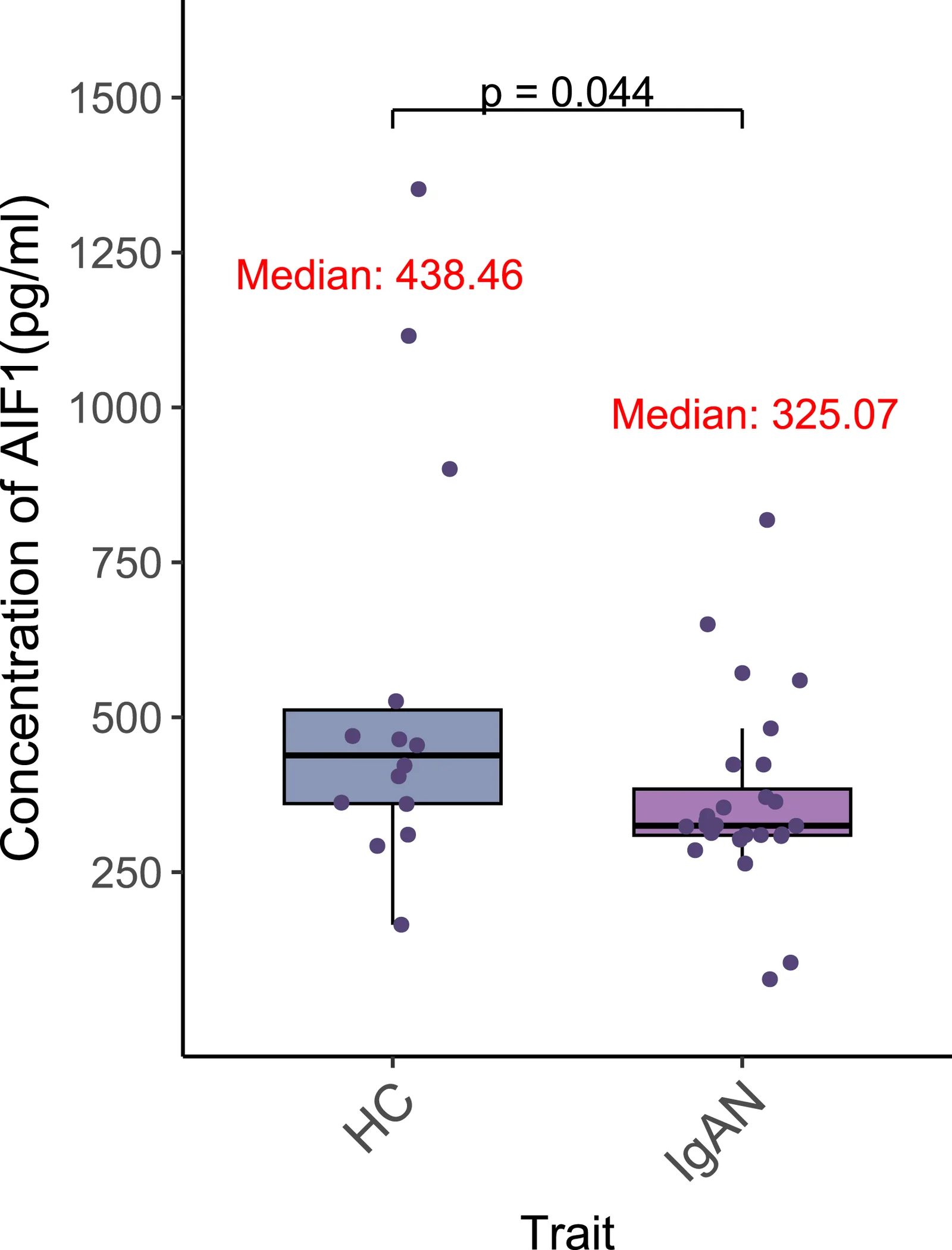
Box plot of differences in plasma AIF1 protein levels between patients with IgA nephropathy (IgAN) and healthy controls, measured using an enzyme-linked immunosorbent assay (ELISA). Each point represents one participant; all 42 samples listed in Supplementary File 2, Table S15 are shown.

## 4 Discussion

This study focused on 25 plasma proteins closely related to IgAN, among which AIF1 was identified as a core hub molecule (Supplementary File 1, Fig. S4). In the Icelandic, Fenland, and the UKB-PPP cohorts, the AIF1 levels all showed a significant negative correlation with IgAN risk, and *AIF1* was downregulated in PBMCs. In patients with IgAN, AIF1 expression levels increased in dendritic cells after tonsillectomy, suggesting that AIF1 may affect disease progression by regulating antigen presentation. Colocalization analysis indicated that the AIF1 pQTL signal and the IgAN GWAS signal map to the same genomic region, although the low posterior probability of a shared causal variant (PP.H4) suggests that the two signals are likely driven by distinct variants. Single-cell communication analysis revealed that lower AIF1 expression was associated with a significant reduction in signal transmission from dendritic cells to CD4^+^ and CD8^+^ T cells. Increased AIF1 levels were associated with a reduced risk of hematuria and albuminuria. Hematuria and albuminuria are the most common early clinical manifestations of IgAN, making AIF1 a promising potential therapeutic target.

Increased AIF1 levels were, however, associated with lower eGFR. This apparent inconsistency may reflect two features of the outcome data. First, the kidney function GWAS were conducted in general population samples, in which IgAN accounts for a negligible fraction of cases, so that the genetic determinants of eGFR in these datasets largely reflect processes unrelated to IgA-mediated glomerular injury. Second, creatinine- and cystatin C-based eGFR are not specific measures of glomerular injury: AIF1 is expressed predominantly in cells of the monocyte-macrophage lineage, and systemic inflammatory state influences both creatinine generation and cystatin C production, so that genetically predicted AIF1 may act on these estimating equations through pathways independent of glomerular filtration itself. The magnitudes of the two sets of associations also differ markedly: AIF1 was associated with hematuria with β = −0.667 and −0.602 in two independent outcome datasets, whereas the corresponding estimates for eGFRcrea were β = −0.011 in both datasets in which it was assessed. These estimates are on different scales and are not directly comparable, but the protective association of AIF1 is most clearly expressed in the direct clinical manifestations of glomerular injury in IgAN. Its relationship with downstream kidney function remains uncertain and requires confirmation in IgAN-specific cohorts.

Among the proteins validated as significant in at least two exposure factor datasets, FCGR3B, DPEP1, BMPER, and S100A12 have strong biological significance. Elevated levels of FCGR3B have been shown to have a synergistic effect with the complement activation marker C4a in autoimmune diseases [38]. FCGR3B is highly expressed in specific subtypes of myeloid leukocytes [39], which might exacerbate glomerular injury by promoting immune complex deposition and complement activation. DPEP1 has been shown to be involved in the degradation of leukotriene D4 and to increase the expression of E-cadherin [40]. Leukotrienes are pro-inflammatory mediators, suggesting that DPEP1 affects IgAN progression through immune regulation. S100A12 was shown to have protective effect on IgAN in all three plasma protein cohorts, which is consistent with its mechanism of regulating neutrophil infiltration through calcium ion signaling and it has been reported to be associated with vascular microcalcification and inflammatory processes [41]. BMPER has been shown to inhibit TGF-β 1-induced tubular dedifferentiation and fibroblast activation, thereby suppressing the progression of chronic kidney disease [42], consistent with the protective effect demonstrated in this study.

Among the significantly colocalized molecular groups, MICA and TAPBP were particularly critical. The significant postoperative increase in MICA in NK cells may be mediated by NKG2D receptors to activate innate immunity [43], whereas TAPBP is involved in HLA-I cell surface localization and is associated with antigen processing and presentation, suggesting that it may play a role in the immune regulatory stage of IgAN progression [44].

The proteome and transcriptome results for B3GAT3 were contradictory. The proteome results suggested that B3GAT3 exhibits a significant protective effect, whereas the transcriptome results suggested that B3GAT3 is associated with a significant increase in risk of IgAN. These contradictory results may be due to feedback regulation of the transcriptome as an upstream process. B3GAT3 is a marker of clear cell renal cell carcinoma and is closely related to B lymphocyte infiltration, providing a possible explanation for the role of B3GAT3 in IgAN progression [45].

MMP7, APOM, and BTN3A3 were also identified as important proteins. By promoting extracellular matrix remodeling, MMP7 is a key driver of fibrosis and is a major biomarker of kidney fibrosis and decline in kidney function [46]. APOM is involved in the APOM/S1P axis, which in turn affects the development of chronic kidney disease [47], and BTN3A3 is associated with a reduced risk of both chronic kidney disease and IgAN [26].

Among the 25 candidate proteins that we selected, the levels of AIF1, FCGR3B, CRP, and MMP7 in plasma were strongly associated with the risk of IgAN, confirming the results of previous studies based on data from other sources [27,48,49]. To our knowledge, the association between the expression levels of 21 other proteins in plasma and the risk of IgAN has not been reported previously.

This study has several limitations. First, no multiple-testing correction was applied at the discovery stage, so the discovery analysis should be regarded as hypothesis-generating; the FDR-corrected replication in the Fenland and UKB-PPP cohorts provides the primary evidence. Second, all exposure cohorts were of European ancestry, so the generalisability of these findings to other populations remains to be established. Third, the ELISA experiment was limited by a small sample size and by a P-value close to the conventional threshold; although the two groups did not differ significantly in age or sex, they were not formally matched and no adjustment for age, sex or kidney function was performed. In addition, the manufacturer did not report intra- or inter-assay coefficients of variation for the ELISA kit used. These results should therefore be regarded as preliminary supporting evidence rather than as independent validation.

## 5 Conclusion

This study identified 25 candidate plasma protein targets for IgAN at the discovery stage, of which 19 were repeatedly validated to have a significant association with IgAN in at least two cohorts, and 8 showed evidence of signal overlap with IgAN at the genomic level. Among the validated plasma protein targets, AIF1 appears to play a core protective role in IgAN and was significantly associated with a reduced risk of IgAN at the proteome, transcriptome, and single-cell transcriptome levels. AIF1 may protect against IgAN by specific expression in dendritic cells, enhancing their antigen presentation ability, and strengthening immune regulation of mucosal infection and autoantigen production.

## CRediT authorship contribution statement

**Xingyu Liu:** Writing – original draft, Visualization, Formal analysis, Data curation, Conceptualization. **Yiyang Hu:** Writing – review & editing, Funding acquisition. **Wenjia Zhang:** Writing – review & editing, Project administration. **Chaohong Nie:** Validation, Investigation. **Fengxia Zhang:** Validation, Investigation. **Yufeng Liu:** Validation. **Xiaoyan Su:** Validation. **Shuangxin Liu:** Supervision, Resources, Conceptualization. **Quhuan Li:** Writing – review & editing, Supervision, Resources, Conceptualization.

## Ethics approval and consent to participate

This study was conducted in accordance with the ethical principles of the Declaration of Helsinki and was performed in compliance with relevant laws, regulatory frameworks and guidelines where the research took place. It was approved by the Ethics Review Committee of the First Affiliated Hospital of Gannan Medical University, China (Approval No. LLSC2023185). Written informed consent to take part in the study and to publish the article was obtained from all participants, or from a parent or legal guardian for participants under 18 years of age, and the privacy rights of participants have been observed. All other data analysed in this study were obtained from publicly available databases and summary-level genome-wide association study datasets, for which ethical approval and participant consent had been obtained in the original studies.

## Funding sources

The present study was funded by the National Natural Science Foundation of China (31870928, 32271360 and 82670992), the Natural Science Foundation of Guangdong Province, China (2023A1515010829), the Guangdong Provincial Basic and Applied Basic Research Fund Project (2025A1515220025), and the Blood Purification Plan of Guangdong Weiji Medical Development Foundation (K-20240103, K-202505-1-13 and K-202505-1-A). The funders had no role in the study design, data collection and analysis, decision to publish, or preparation of the manuscript.

## Declaration of competing interest

The authors declare that they have no known competing financial interests or personal relationships that could have appeared to influence the work reported in this paper.

## Figures and Tables

**Table:** 

AIF1	Allograft inflammatory factor 1
APOM	Apolipoprotein M
B3GAT3	Galactosyl galactosyl xylosyl protein 3-beta-glucuronosyltransferase 3
BMPER	BMP-binding endothelial regulator protein
BTN3A3	Butyrophilin subfamily 3 member A3
CD177	CD177 antigen
CI	confidence interval
CLIC5	Chloride intracellular channel protein 5
CRP	C-reactive protein
DCs	dendritic cells
DPEP1	Dipeptidase 1
eGFR	estimated glomerular filtration rate
EPHB1	Ephrin type-B receptor 1
eQTL	expression quantitative trait loci
F2	Coagulation factor II
FDR	false discovery rate
FCGR3B	Low affinity immunoglobulin gamma Fc region receptor III-B
FLRT3	Fibronectin Leucine Rich Transmembrane Protein 3
Gd-IgA1	Galactose-deficient IgA1
GEO	Gene Expression Omnibus
GMPR2	GMP reductase 2
GO	Gene Ontology
GWAS	Genome-wide association study
HBD	Healthy blood donors
HEBP1	Heme-binding protein 1
ICD	International Classification of Diseases
IgAN	IgA nephropathy
IVs	instrumental variables
IVW	Inverse variance weighted
LD	Linkage disequilibrium
LIMA1	LIM domain and actin-binding protein 1
MICA	MHC class I polypeptide-related sequence A
MMP7	matrix metallopeptidase 7
MR	Mendelian randomization
MR-PRESSO	Mendelian Randomization Pleiotropy RESidual Sum and Outlier
NK	natural killer
PBMC	peripheral blood mononuclear cell
PPI	Protein-protein interaction
pQTL	protein quantitative trait loci
PWAS	Proteome-wide association study
RAB31	Ras-related protein Rab-31
RAB6B	Ras-related protein Rab-6B
S100A12	S100 calcium binding protein A12
sceQTL	Single-cell expression quantitative trait loci
scTWAS	Single-cell transcriptome-wide association study
SE	standard error
SNP	Single nucleotide polymorphism
SRA1	Steroid receptor RNA activator 1
TAPBP	tapasin

## Data Availability

All datasets analysed in this study are openly available in public repositories. Plasma proteomic genetic association data were obtained from deCODE genetics, the Fenland study and the UK Biobank Pharma Proteomics Project; genome-wide association summary statistics for IgA nephropathy, kidney function and clinical phenotypes were obtained from the GWAS Catalog and the IEU OpenGWAS database; transcriptomic and single-cell transcriptomic data were obtained from the Gene Expression Omnibus (accession numbers GSE58539 and GSE189481); and expression quantitative trait locus data were obtained from the eQTLGen Consortium and OneK1K. Accession numbers and access details for each dataset are given in the Methods section. Individual-level ELISA measurements generated in this study are provided in Supplementary File 2 (Table S15); any further data are available from the corresponding author on reasonable request.
