# Supplementary material for: Integrating multiple omics approaches to identify AIF1 as a protective factor in IgA nephropathy
Source: J Transl Autoimmun. 2026 Oct 7;13:100407. doi: 10.1016/j.jtauto.2026.100407 (PMC13678103; doi:10.1016/j.jtauto.2026.100407)
Supplement: Supplementary file 1 [file mmc1.pdf]

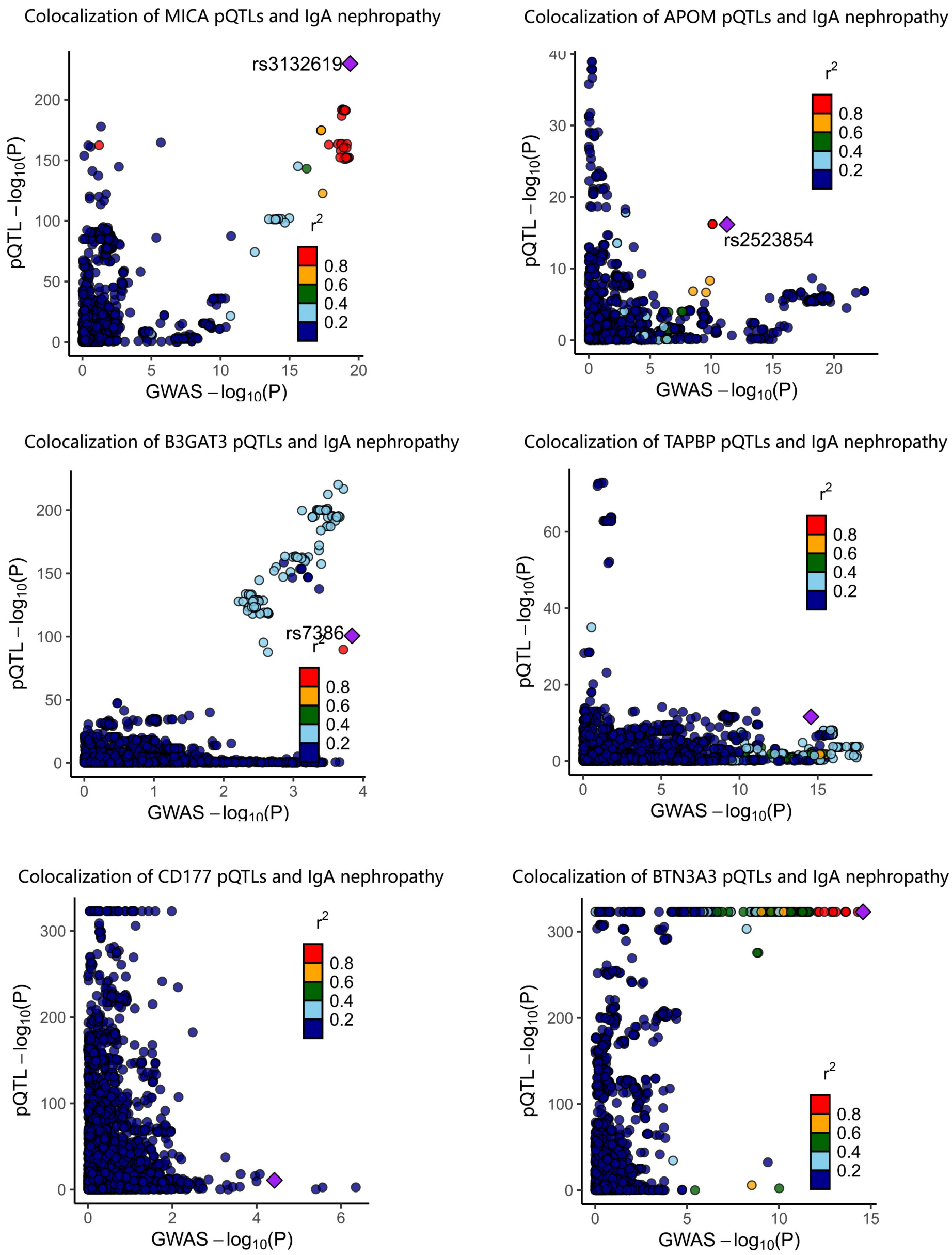

Figure S1. Colocalization results of six primary candidate plasma protein molecules: MICA, APOM, B3GAT3, TAPBP, CD177, and BTN3A3.

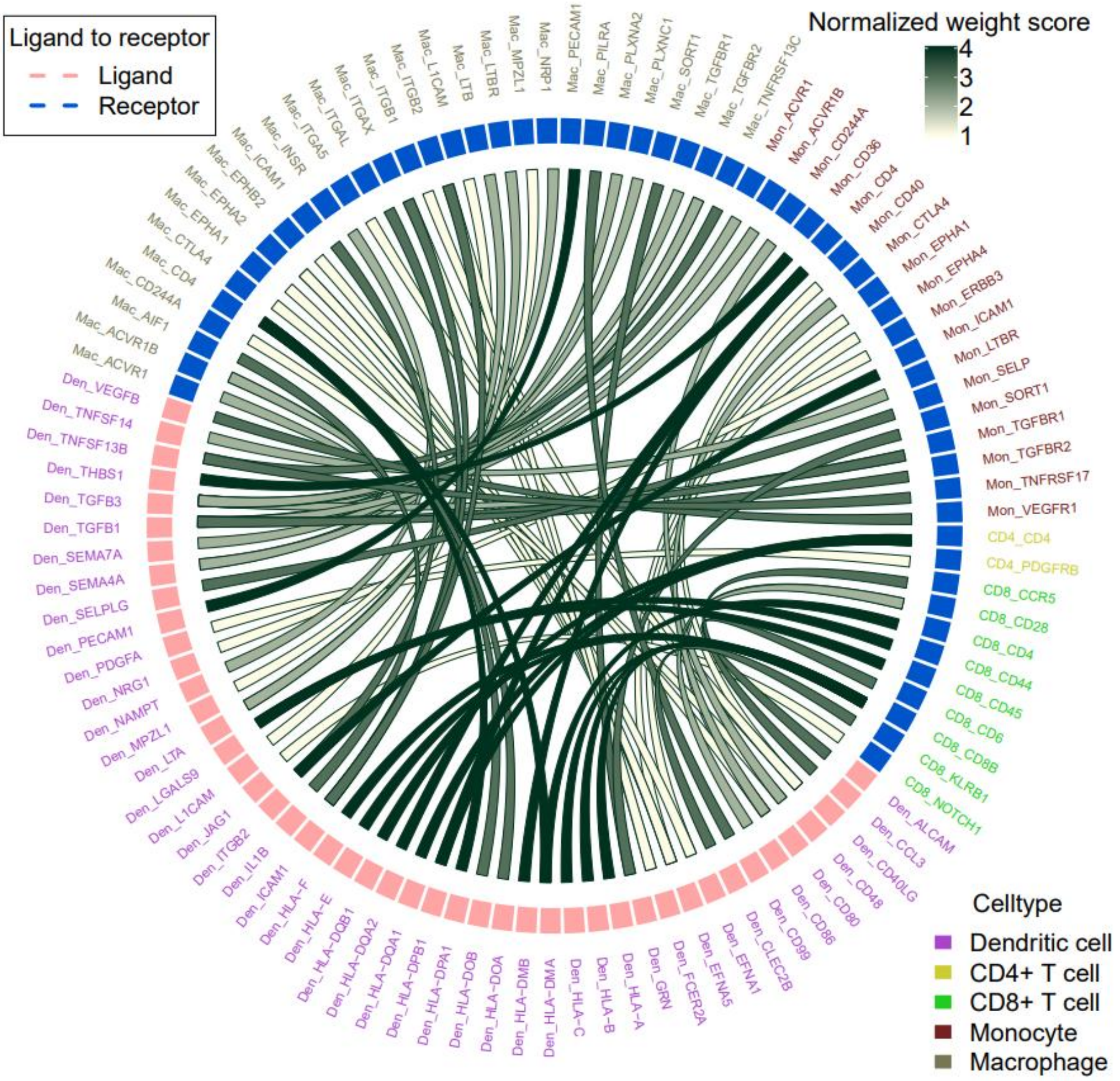

Fig. S2. Chord diagram of ligand–receptor interactions between dendritic cells (Den) and CD4+ T cells (CD4), CD8+ T cells (CD8), monocytes (Mon), and macrophages (Mac) in patients with IgA nephropathy (IgAN) at 150 days after tonsillectomy. Outer arc segments are grouped by cell type and colored to indicate ligand or receptor status; line color and thickness represent the normalized interaction weight score (scale, 1–4).

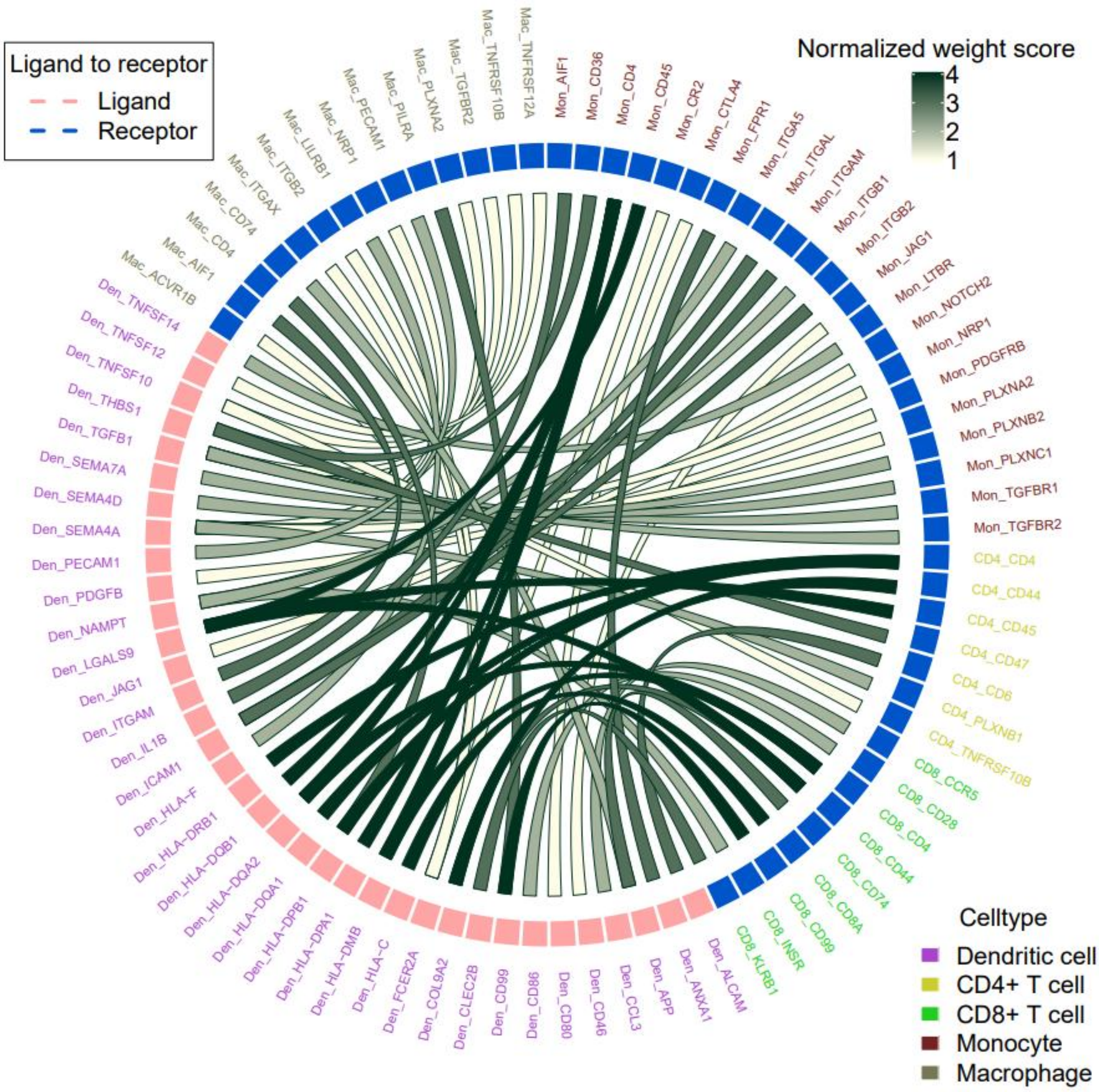

Fig. S3. Chord diagram of ligand–receptor interactions between dendritic cells (Den) and CD4+ T cells (CD4), CD8+ T cells (CD8), monocytes (Mon), and macrophages (Mac) in patients with IgA nephropathy (IgAN) before tonsillectomy. Outer arc segments are grouped by cell type and colored to indicate ligand or receptor status; line color and thickness represent the normalized interaction weight score (scale, 1–4).

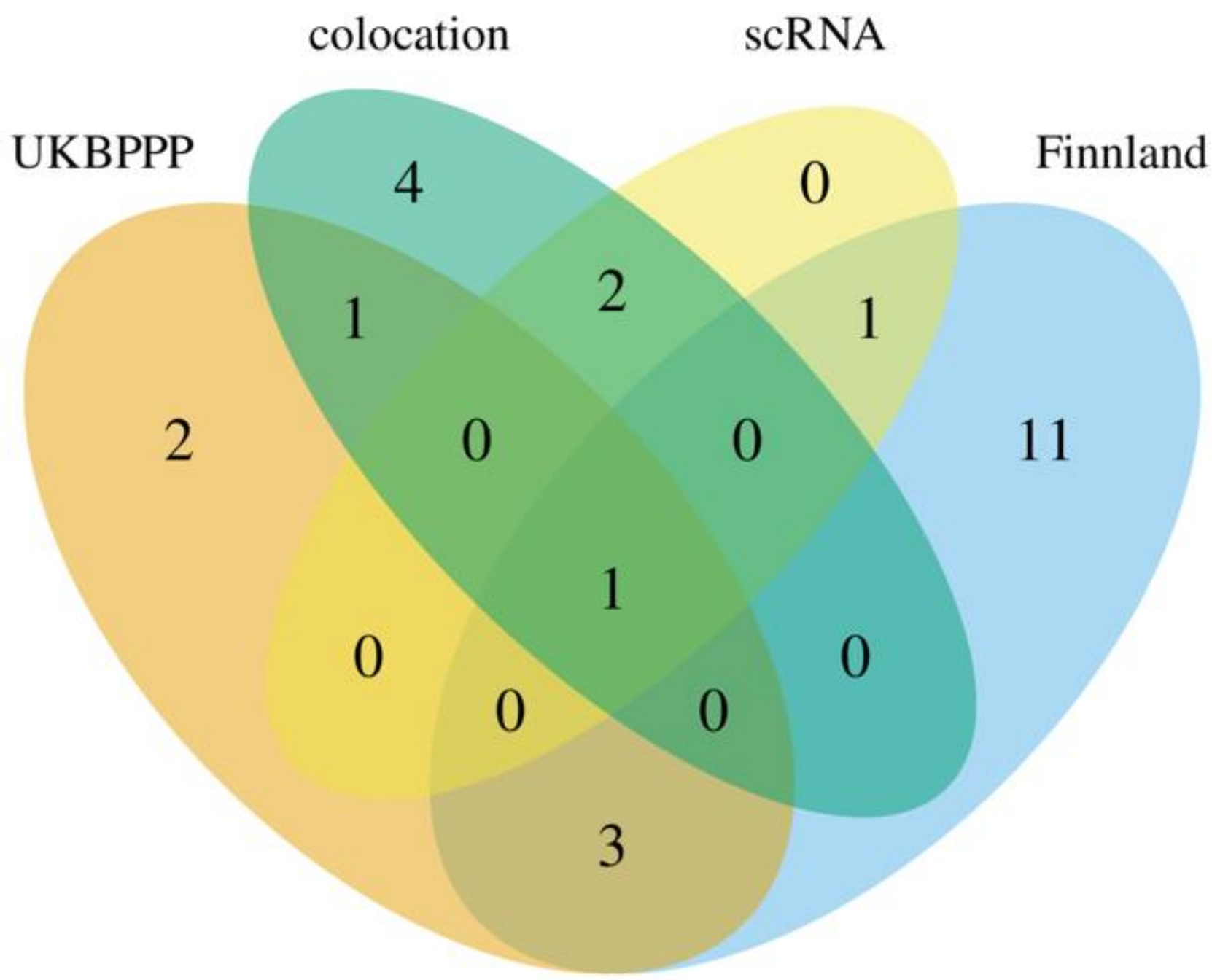

Figure S4. Based on 92 primary candidate plasma proteins, the Venn plots of plasma protein molecules which passed the following tesets such as UKB-PPP dataset rescreening, Finnish dataset rescreening, colocalization analysis, and significant differences between single-cell subtype. AIF1 showed significant results in all four verifications.
