# Supplementary material for: Integrating multiple omics approaches to identify AIF1 as a protective factor in IgA nephropathy
Source: J Transl Autoimmun. 2026 Oct 7;13:100407. doi: 10.1016/j.jtauto.2026.100407 (PMC13678103; doi:10.1016/j.jtauto.2026.100407)
Supplement: Supplementary file 2 [file mmc2.docx]

**STROBE-MR checklist of recommended items to address in reports of Mendelian randomization studies**^1^ ^2^

| **Item No.** | **Section** | **Checklist item** | **Page No.** | **Relevant text from manuscript** |
| --- | --- | --- | --- | --- |
| 1 | **TITLE and ABSTRACT** | Indicate Mendelian randomization (MR) as the study’s design in the title and/or the abstract if that is a main purpose of the study | 1 | **Integrating multiple omics approaches to identify AIF1 as a protective factor in IgA nephropathy**  **IgA nephropathy (IgAN) is the most prevalent primary glomerular disease worldwide, and its diagnosis still relies on invasive kidney biopsy. To identify noninvasive biomarkers and therapeutic targets, we applied a multi-ancestry Mendelian randomization (MR) framework using proteome-wide association data from an Icelandic population, with validation in independent Fenland and United Kingdom cohorts, followed by transcriptomic, single-cell RNA sequencing (scRNA-seq), and cell–cell communication analyses. Nineteen candidate proteins were identified, and Bayesian colocalization supported a shared genomic region for eight, including AIF1. Transcriptomic analysis revealed downregulation of AIF1 in peripheral blood mononuclear cells from patients with IgAN, and scRNA-seq localized this depletion to dendritic cells (DCs), with partial restoration 150 days after tonsillectomy. Clinical phenotype MR showed inverse associations between AIF1 and hematuria and albuminuria, and enzyme-linked immunosorbent assay (ELISA) showed reduced circulating AIF1 in patients. AIF1 may protect against IgAN through DC-mediated antigen presentation, representing a candidate noninvasive biomarker and therapeutic target.** |
|  | **INTRODUCTION** |  | 2 -3 |  |
| 2 | **Background** | Explain the scientific background and rationale for the reported study. What is the exposure? Is a potential causal relationship between exposure and outcome plausible? Justify why MR is a helpful method to address the study question | 2 | Immunoglobulin A (IgA) nephropathy (IgAN) is the most common primary glomerular disease worldwide. It is characterized by the deposition of IgA-formed immune complexes in the mesangial regions of the kidney. The disease has a spectrum of clinical manifestations, ranging from asymptomatic hematuria and proteinuria to progressive impairment of kidney function, ultimately leading to kidney failure. The pathophysiology of IgAN is complex and multifactorial, and involves genetic predisposition, environmental triggers, and dysregulated immune responses that contribute to aberrant IgA production and subsequent glomerular deposition [1,2]. The systemic immune environment in the blood acts as a crucial upstream trigger that precipitates the subsequent local kidney tissue injury [3,4].  Despite advances in the understanding of the disease, current diagnostic strategies for IgAN rely largely on kidney biopsy, which provides histopathological confirmation but carries a risk of complications and does not reflect the systemic nature of the disease. Therefore, noninvasive biomarkers that facilitate early diagnosis and can be used to monitor disease progression, without the inherent risks associated with invasive procedures, are urgently needed. Recent studies have highlighted the potential of urinary metabolites and serum proteins as biomarkers of IgAN [5,6], but these findings have not been translated into clinical practice owing to their lack of specificity and sensitivity.  Considering these challenges, this study aimed to leverage Mendelian randomization (MR) as an analytical framework to elucidate the causal relationships between various biological exposures and IgAN outcomes [7]. MR uses genetic variants as instrumental variables (IVs) to circumvent confounding and rule out reverse causation, thereby providing stronger evidence for causal inference. By integrating proteomic and transcriptomic data through MR analysis, this study aimed to identify and validate novel plasma protein biomarkers associated with risk of IgAN development and progression. |
| 3 | **Objectives** | State specific objectives clearly, including pre-specified causal hypotheses (if any). State that MR is a method that, under specific assumptions, intends to estimate causal effects | 2-3 | Despite advances in the understanding of the disease, current diagnostic strategies for IgAN rely largely on kidney biopsy, which provides histopathological confirmation but carries a risk of complications and does not reflect the systemic nature of the disease. Therefore, noninvasive biomarkers that facilitate early diagnosis and can be used to monitor disease progression, without the inherent risks associated with invasive procedures, are urgently needed. Recent studies have highlighted the potential of urinary metabolites and serum proteins as biomarkers of IgAN [5,6], but these findings have not been translated into clinical practice owing to their lack of specificity and sensitivity.  Considering these challenges, this study aimed to leverage Mendelian randomization (MR) as an analytical framework to elucidate the causal relationships between various biological exposures and IgAN outcomes [7]. MR uses genetic variants as instrumental variables (IVs) to circumvent confounding and rule out reverse causation, thereby providing stronger evidence for causal inference. By integrating proteomic and transcriptomic data through MR analysis, this study aimed to identify and validate novel plasma protein biomarkers associated with risk of IgAN development and progression. |
|  | **METHODS** |  | 3-10 |  |
| 4 | **Study design and data sources** | Present key elements of the study design early in the article. Consider including a table listing sources of data for all phases of the study. For each data source contributing to the analysis, describe the following: | 3-7 |  |
|  | a) | Setting: Describe the study design and the underlying population, if possible. Describe the setting, locations, and relevant dates, including periods of recruitment, exposure, follow-up, and data collection, when available. | 3 | The analysis pipeline was defined as follows (Fig. 1): (1) A two-sample MR method was used to select primary potential plasma protein targets for IgAN from a cohort of 35,599 Icelandic individuals through PWAS. Repeated validation was conducted on European cohorts of 54,219 individuals in the UK Biobank (UKB) and a cohort of 10,708 Fenland individuals. (2) Protein quantitative trait loci (pQTLs) of primary candidate plasma proteins were selected and colocalization analysis was performed with IgAN and the most statistically significant single-nucleotide polymorphisms (SNPs) (with the least P) to explore whether the SNP levels and IgAN were affected by the same variation within a certain range. (3) The expression of candidate plasma proteins in peripheral blood mononuclear cells (PBMCs) was verified using Gene Expression Omnibus (GEO, https://www.ncbi.nlm.nih.gov/gds/) datasets and a transcriptome-wide association study (TWAS) of candidate proteins was conducted with an expression quantitative trait locus (eQTL) to validate the association between candidate proteins and IgAN. (4) To verify the association between the expression of candidate proteins in specific cell subtypes and IgAN, a GEO single-cell transcriptome sequencing dataset was used to identify potential cell subtypes carrying the candidate proteins using single-cell specific eQTL (sceQTL) data from OneK1K (https://onek1k.org/). (5) Participation of the candidate protein targets in intercellular interactions in specific cell subtypes was verified through cell-communication analysis to reveal the cellular interactions of patients before and after tonsillectomy. (6) The effects of candidate protein targets were validated by MR replication analysis of common clinical manifestations of IgAN and kidney function. (7) Potential biological mechanisms for inferring protein targets were explored through protein-protein interaction (PPI) and Gene Ontology (GO) enrichment analysis. (8) Plasma levels of the target proteins in patients with IgAN and healthy controls were compared using enzyme-linked immunosorbent assays (ELISA). |
|  | b) | Participants: Give the eligibility criteria, and the sources and methods of selection of participants. Report the sample size, and whether any power or sample size calculations were carried out prior to the main analysis | 3-7 | The exposure data were derived from summary statistics of three large GWAS datasets: (1) Plasma protein genetic associations were obtained from deCODE genetics (Reykjavik, Iceland). A large-scale proteomic study was conducted of 40,004 Icelanders (52% from the Icelandic Cancer Project and 48% from various genetic programs at deCODE Genetics, Reykjavik) [8]. A total of 35,559 blood samples were selected to measure protein levels using SomaScan version 4 (SomaLogic, Boulder, CO, USA), which identified 28,191 genetic associations (P < 1.8 × 10⁻⁹). (2) A total of 10,674 genetic associations (8,328 sentinel signals and 2,346 secondary signals, P < 1.004 × 10−11) for 3,892 plasma proteins identified in 10,708 participants in the Fenland cohort (Cambridgeshire, UK)[9]. (3) A total of 23,588 sentinel genetic associations (P < 1.7 × 10−11) for 2,923 plasma proteins were identified in 54,219 participants from the UK Biobank Pharma Proteomics Project (UKB-PPP) using the Olink platform [10]. Cis-pQTLs were defined as genetic variants within 1 Mb upstream or downstream of the transcription start site of the protein-encoding gene, whereas trans-pQTLs were defined as variants that influenced protein levels but exceeded the cis-regulatory region. |
|  | c) | Describe measurement, quality control and selection of genetic variants | 6 | We used a two-sample MR approach to identify potential plasma protein targets for IgAN [22]. The analysis was conducted based on the three core assumptions of MR (Fig. 1): (1) The IVs must be strongly associated with the exposure; (2) The IVs must influence the outcome exclusively through the exposure; and (3) The IVs must be independent of any confounding factors which may also influence the outcome. To ensure that these assumptions were met, we implemented the following measures: We restricted our IVs to cis-pQTLs with P < 5×10⁻⁸ and calculated the F-statistic for each cis-pQTL to evaluate its strength. For a single pQTL, the F-statistic was approximated as: F=β2SE2 [23], where β is the effect of the pQTL on protein levels, and SE is the standard error of the association [22]. We filtered out IVs with F-statistic values <10 and used the ld_clump function from the ‘ieugwasr’ package to filter pQTLs in the European reference genome with parameters clump_kb = 10000 and clump_r2 = 0.001, to control for the influence of linkage disequilibrium (LD) [19]. |
|  | d) | For each exposure, outcome, and other relevant variables, describe methods of assessment and diagnostic criteria for diseases | NA |  |
|  | e) | Provide details of ethics committee approval and participant informed consent, if relevant | 7 | The study was approved by the Ethics Review Committee of the First Affiliated Hospital of Gannan Medical University (approval number LLSC2023185). Written informed consent was obtained from all participants, or from a parent or legal guardian for participants under 18 years of age, and the study was conducted in accordance with the Declaration of Helsinki. (Section 2.10) The Mendelian randomization analyses used publicly available summary-level GWAS data, for which ethical approval had been obtained in the original studies. |
| 5 | **Assumptions** | Explicitly state the three core IV assumptions for the main analysis (relevance, independence and exclusion restriction) as well assumptions for any additional or sensitivity analysis | 3 | The analysis pipeline was defined as follows (Fig. 1): (1) A two-sample MR method was used to select primary potential plasma protein targets for IgAN from a cohort of 35,599 Icelandic individuals through PWAS. Repeated validation was conducted on European cohorts of 54,219 individuals in the UK Biobank (UKB) and a cohort of 10,708 Fenland individuals |
| 6 | **Statistical methods: main analysis** | Describe statistical methods and statistics used | 5-9 |  |
|  | a) | Describe how quantitative variables were handled in the analyses (i.e., scale, units, model) | 5 | The EGFRcrea data were winsorized at 15 and 200 mL/min/1.73 m2 [16] |
|  | b) | Describe how genetic variants were handled in the analyses and, if applicable, how their weights were selected | 6-9 | We used a two-sample MR approach to identify potential plasma protein targets for IgAN [22]. The analysis was conducted based on the three core assumptions of MR (Fig. 1): (1) The IVs must be strongly associated with the exposure; (2) The IVs must influence the outcome exclusively through the exposure; and (3) The IVs must be independent of any confounding factors which may also influence the outcome. To ensure that these assumptions were met, we implemented the following measures: We restricted our IVs to cis-pQTLs with P < 5×10⁻⁸ and calculated the F-statistic for each cis-pQTL to evaluate its strength. For a single pQTL, the F-statistic was approximated as: F=β2SE2 [23], where β is the effect of the pQTL on protein levels, and SE is the standard error of the association [22]. We filtered out IVs with F-statistic values <10 and used the ld_clump function from the ‘ieugwasr’ package to filter pQTLs in the European reference genome with parameters clump_kb = 10000 and clump_r2 = 0.001, to control for the influence of linkage disequilibrium (LD) [19]. |
|  | c) | Describe the MR estimator (e.g. two-stage least squares, Wald ratio) and related statistics. Detail the included covariates and, in case of two-sample MR, whether the same covariate set was used for adjustment in the two samples | 6 | For proteins with a single cis-pQTL, the Wald ratio method was used to estimate the causal effect size (β) and 95% corresponding confidence interval (CI) [24], whereas for proteins with multiple cis-pQTLs, estimates were obtained using the inverse-variance weighted (IVW) method [25] |
|  | d) | Explain how missing data were addressed | NA |  |
|  | e) | If applicable, indicate how multiple testing was addressed | 6 | Repeated MR analysis of the primary candidate plasma proteins were subsequently performed using the Fenland and UKB-PPP cohorts as replication datasets and the results were corrected for the False Discovery Rate (FDR). |
| 7 | **Assessment of assumptions** | Describe any methods or prior knowledge used to assess the assumptions or justify their validity | 6-9 | To ensure that these assumptions were met, we implemented the following measures: We restricted our IVs to cis-pQTLs with P < 5×10⁻⁸ and calculated the F-statistic for each cis-pQTL to evaluate its strength. For a single pQTL, the F-statistic was approximated as: F=β2SE2 [23], where β is the effect of the pQTL on protein levels, and SE is the standard error of the association [22]. We filtered out IVs with F-statistic values <10 and used the ld_clump function from the ‘ieugwasr’ package to filter pQTLs in the European reference genome with parameters clump_kb = 10000 and clump_r2 = 0.001, to control for the influence of linkage disequilibrium (LD) [19]. |
| 8 | **Sensitivity analyses and additional analyses** | Describe any sensitivity analyses or additional analyses performed (e.g. comparison of effect estimates from different approaches, independent replication, bias analytic techniques, validation of instruments, simulations) | 7 | The MR-Egger method was used to test for directional (horizontal) pleiotropy [28]. We tested that the MR-Egger regression intercept had a threshold P-value > 0.05 to rule out the presence of significant horizontal pleiotropy. A nonsignificant intercept term indicates that the horizontal pleiotropy of the IVs is within a controllable range. |
| 9 | **Software and pre-registration** |  | 7-9 |  |
|  | a) | Name statistical software and package(s), including version and settings used | 7-9 | The MR and sensitivity analyses were performed using ‘TwoSampleMR’ R package (version 0.6.8) [19]. |
|  | b) | State whether the study protocol and details were pre-registered (as well as when and where) | NA | This study was not pre-registered. |
|  | **RESULTS** |  | 9-15 |  |
| 10 | **Descriptive data** |  |  |  |
|  | a) | Report the numbers of individuals at each stage of included studies and reasons for exclusion. Consider use of a flow diagram | 9 | In the Icelandic discovery cohort, 92 of the 1,763 plasma proteins with valid instruments after LD clumping were significantly associated with IgAN (Fig. 2A). Detailed data on these associations in the discovery datasets and the results of the sensitivity analysis are shown in Supplementary File 2, Table S3. |
|  | b) | Report summary statistics for phenotypic exposure(s), outcome(s), and other relevant variables (e.g. means, SDs, proportions) | 10-15 | Among these plasma proteins, AIF1, BMPER, DPEP1, and S100A12 were validated as significant in all three cohorts. Their β values (95% CIs) were −0.584 (−0.910 to −0.257), 0.213 (0.010 to 0.415), −0.075 (−0.138 to −0.012) and −0.136 (−0.243 to −0.030) in the discovery dataset. |
|  | c) | If the data sources include meta-analyses of previous studies, provide the assessments of heterogeneity across these studies | 11，14-15 | Fifteen proteins, including FCGR3B, LIMA1, MMP7, RAB31, RAB6B, and SRA1 were validated as significant in two of the three cohorts. Their β values (95% CIs) were 0.151 (0.092 to 0.210) for FCGR3B, 0.410 (0.021 to 0.799) for LIMA1, 0.090 (0.011 to 0.169) for MMP7, 0.269 (0.095 to 0.443) for RAB31, −0.132 (−0.252 to −0.012) for RAB6B, and 0.292 (0.094 to 0.491) and SRA1. |
|  | d) | For two-sample MR:  i.  Provide justification of the similarity of the genetic variant-exposure associations between the exposure and outcome samples  ii.  Provide information on the number of individuals who overlap between the exposure and outcome studies | NA |  |
| 11 | **Main results** |  | 9-15 |  |
|  | a) | Report the associations between genetic variant and exposure, and between genetic variant and outcome, preferably on an interpretable scale | NA |  |
|  | b) | Report MR estimates of the relationship between exposure and outcome, and the measures of uncertainty from the MR analysis, on an interpretable scale, such as odds ratio or relative risk per SD difference | 9-15 | Among these plasma proteins, AIF1, BMPER, DPEP1, and S100A12 were validated as significant in all three cohorts. Their β values (95% CIs) were −0.584 (−0.910 to −0.257), 0.213 (0.010 to 0.415), −0.075 (−0.138 to −0.012) and −0.136 (−0.243 to −0.030) in the discovery dataset. Fifteen proteins, including FCGR3B, LIMA1, MMP7, RAB31, RAB6B, and SRA1 were validated as significant in two of the three cohorts. Their β values (95% CIs) were 0.151 (0.092 to 0.210) for FCGR3B, 0.410 (0.021 to 0.799) for LIMA1, 0.090 (0.011 to 0.169) for MMP7, 0.269 (0.095 to 0.443) for RAB31, −0.132 (−0.252 to −0.012) for RAB6B, and 0.292 (0.094 to 0.491) and SRA1. |
|  | c) | If relevant, consider translating estimates of relative risk into absolute risk for a meaningful time period | NA |  |
|  | d) | Consider plots to visualize results (e.g. forest plot, scatterplot of associations between genetic variants and outcome versus between genetic variants and exposure) | 9-15 | In addition, in the discovery dataset six other plasma proteins, MICA, APOM, TAPBP, CD177, B3GAT3, and BTN3A3, colocalized with IgAN. Their β values (95% CIs) were −0.14 (−0.21 to −0.06) for MICA, −0.22 (−0.42 to −0.02) for APOM, −0.13 (−0.22 to −0.04) for TAPBP, 0.04 (0.00 to 0.08) for CD177, −0.16 (−0.25 to −0.07) for B3GAT3, and 0.09 (0.00 to 0.17) for BTN3A3 (Supplementary File 2, Table S3). Fig. 2D shows a summary of the associations between these 25 plasma proteins and IgAN in the three cohorts. |
| 12 | **Assessment of assumptions** |  | 9-15 |  |
|  | a) | Report the assessment of the validity of the assumptions | 9-12，14-15 | In this study, the Cochran Q-test indicated no significant heterogeneity, and the fixed-effects IVW model was therefore used. All 25 proteins showed nominally significant associations with IgAN in at least one cohort, and were carried forward as candidate targets for downstream validation. |
|  | b) | Report any additional statistics (e.g., assessments of heterogeneity across genetic variants, such as *I^2^*, Q statistic or E-value) | 12，14-15 | The scTWAS results of the associations of proteins with significant expression differences between specific cell subtypes and IgAN showed that the AIF1 expression levels in dendritic cells estimated were consistently negatively correlated with IgAN using the five MR methods (Fig. 5A). The β (95% CI) were: −0.049 (−0.105 to 0.006) for Weighted median, −0.047 (−0.090 to −0.005) for IVW, −0.050 (−0.125 to 0.024) for Simple mode, −0.050 (−0.123 to 0.023) for Weighted mode, and −0.047 (−0.054 to −0.041) for MR-PRESSO (Supplementary File 2, Table S8). The P-values for the IVW method and MR-PRESSO were 0.02875 and 6.87E−7, respectively. The Cochrane’s Q-test did not reveal any significant heterogeneity, and in the MR-PRESSO test did not reveal any obvious outliers or pleiotropy. |
| 13 | **Sensitivity analyses and additional analyses** |  | 9-15 |  |
|  | a) | Report any sensitivity analyses to assess the robustness of the main results to violations of the assumptions | 10 | Although MICA, B3GAT3, APOM, TAPBP, CD177, and BTN3A3 were validated only in the MR of the Icelandic data, their positive colocalization indicated their potential as IgAN targets (Fig. 2E and Supplementary File 1, Fig. S1). As a negative colocalization result does not necessarily invalidate the MR results, an additional 6 proteins were included along with the 19 previously identified proteins to explore candidate targets. |
|  | b) | Report results from other sensitivity analyses or additional analyses | 6 | “This study used the Cochran Q-test to evaluate heterogeneity… The MR-Egger method was used to test for directional (horizontal) pleiotropy. We tested that the MR-Egger regression intercept had a threshold P-value > 0.05 to rule out the presence of significant horizontal pleiotropy.” (Section 2.5) Heterogeneity and pleiotropy statistics for every exposure are reported in Supplementary File 2, Tables S3 and S4 (columns Q_pval and Pleio). For the single-cell TWAS, five estimators (IVW, weighted median, simple mode, weighted mode and MR-PRESSO) were applied and gave consistent estimates (Table S8). |
|  | c) | Report any assessment of direction of causal relationship (e.g., bidirectional MR) | 9-15 | Fifteen proteins, including FCGR3B, LIMA1, MMP7, RAB31, RAB6B, and SRA1 were validated as significant in two of the three cohorts. Their β values (95% CIs) were 0.151 (0.092 to 0.210) for FCGR3B, 0.410 (0.021 to 0.799) for LIMA1, 0.090 (0.011 to 0.169) for MMP7, 0.269 (0.095 to 0.443) for RAB31, −0.132 (−0.252 to −0.012) for RAB6B, and 0.292 (0.094 to 0.491) and SRA1. |
|  | d) | When relevant, report and compare with estimates from non-MR analyses | 12-15 | The MR results were compared with non-MR evidence obtained in the same study: “AIF1 expression was significantly downregulated in PBMCs of patients with IgAN (logFC = −0.156, P = 0.036)” (Section 3.2, GSE58539); single-cell RNA sequencing before and after tonsillectomy (Section 3.3, GSE189481); and “median plasma AIF1 concentration was 438.46 pg/mL in healthy controls and 325.07 pg/mL in patients with IgAN (P = 0.044, Mann–Whitney U-test)” (Section 3.7, ELISA). All three were concordant in direction with the MR estimates. |
|  | e) | Consider additional plots to visualize results (e.g., leave-one-out analyses) | 12-13 | The differential analysis of the genes encoding the candidate proteins expressed in various cell subtypes of patients with IgAN before and 150 days after tonsillectomy showed that the preoperative and postoperative expression levels did not differ significantly in most cell subtypes. However, the expression levels of AIF1, MICA, TAPBP, and SRA1 differed significantly in specific cell subtypes (Fig. 4B,C). |
|  | **DISCUSSION** |  | 16-18 |  |
| 14 | **Key results** | Summarize key results with reference to study objectives | 16 | This study focused on 25 plasma proteins closely related to IgAN, among which AIF1 was identified as a core hub molecule (Supplementary File 1, Fig. S4). In the Icelandic, Fenland, and the UKB-PPP cohorts, the AIF1 levels all showed a significant negative correlation with IgAN risk, and AIF1 was downregulated in PBMCs. In patients with IgAN, AIF1 expression levels increased in dendritic cells after tonsillectomy, suggesting that AIF1 may affect disease progression by regulating antigen presentation. Colocalization analysis indicated that the AIF1 pQTL signal and the IgAN GWAS signal map to the same genomic region, although the low posterior probability of a shared causal variant (PP.H4) suggests that the two signals are likely driven by distinct variants. Single-cell communication analysis revealed that lower AIF1 expression was associated with a significant reduction in signal transmission from dendritic cells to CD4+ and CD8+ T cells. Increased AIF1 levels were associated with a reduced risk of hematuria and albuminuria. Hematuria and albuminuria are the most common early clinical manifestations of IgAN, making AIF1 a promising potential therapeutic target. |
| 15 | **Limitations** | Discuss limitations of the study, taking into account the validity of the IV assumptions, other sources of potential bias, and imprecision. Discuss both direction and magnitude of any potential bias and any efforts to address them | 18 | This study has several limitations. First, no multiple-testing correction was applied at the discovery stage, so the discovery analysis should be regarded as hypothesis-generating; the FDR-corrected replication in the Fenland and UKB-PPP cohorts provides the primary evidence. Second, all exposure cohorts were of European ancestry, so the generalisability of these findings to other populations remains to be established. Third, the ELISA experiment was limited by a small sample size and by a P-value close to the conventional threshold; although the two groups did not differ significantly in age or sex, they were not formally matched and no adjustment for age, sex or kidney function was performed. In addition, the manufacturer did not report intra- or inter-assay coefficients of variation for the ELISA kit used. These results should therefore be regarded as preliminary supporting evidence rather than as independent validation. |
| 16 | **Interpretation** |  | 17-18 |  |
|  | a) | Meaning: Give a cautious overall interpretation of results in the context of their limitations and in comparison with other studies | 17-18 | Among the 25 candidate proteins that we selected, the levels of AIF1, FCGR3B, CRP, and MMP7 in plasma were strongly associated with the risk of IgAN, confirming the results of previous studies based on data from other sources [27,48,49]. To our knowledge, the association between the expression levels of 21 other proteins in plasma and the risk of IgAN has not been reported previously. |
|  | b) | Mechanism: Discuss underlying biological mechanisms that could drive a potential causal relationship between the investigated exposure and the outcome, and whether the gene-environment equivalence assumption is reasonable. Use causal language carefully, clarifying that IV estimates may provide causal effects only under certain assumptions | 17-18 | Among the proteins validated as significant in at least two exposure factor datasets, FCGR3B, DPEP1, BMPER, and S100A12 have strong biological significance. Elevated levels of FCGR3B have been shown to have a synergistic effect with the complement activation marker C4a in autoimmune diseases [38]. FCGR3B is highly expressed in specific subtypes of myeloid leukocytes [39], which might exacerbate glomerular injury by promoting immune complex deposition and complement activation. DPEP1 has been shown to be involved in the degradation of leukotriene D4 and to increase the expression of E-cadherin [40]. Leukotrienes are pro-inflammatory mediators, suggesting that DPEP1 affects IgAN progression through immune regulation. S100A12 was shown to have protective effect on IgAN in all three plasma protein cohorts, which is consistent with its mechanism of regulating neutrophil infiltration through calcium ion signaling and it has been reported to be associated with vascular microcalcification and inflammatory processes [41]. BMPER has been shown to inhibit TGF‑β 1-induced tubular dedifferentiation and fibroblast activation, thereby suppressing the progression of chronic kidney disease [42], consistent with the protective effect demonstrated in this study. |
|  | c) | Clinical relevance: Discuss whether the results have clinical or public policy relevance, and to what extent they inform effect sizes of possible interventions | 17-18 | “AIF1 may protect against IgAN by specific expression in dendritic cells, enhancing their antigen presentation ability, and strengthening immune regulation of mucosal infection and autoantigen production.” (Section 5) IgAN currently requires kidney biopsy for diagnosis, so a circulating protein such as AIF1 is of potential clinical relevance as a noninvasive biomarker and as a therapeutic target; confirmation in IgAN-specific cohorts is required before clinical application. |
| 17 | **Generalizability** | Discuss the generalizability of the study results (a) to other populations, (b) across other exposure periods/timings, and (c) across other levels of exposure | 18 | These results should therefore be regarded as preliminary supporting evidence rather than as independent validation. |
|  | **OTHER INFORMATION** |  | 18-21 |  |
| 18 | **Funding** | Describe sources of funding and the role of funders in the present study and, if applicable, sources of funding for the databases and original study or studies on which the present study is based | 20 | The present study was funded by the National Natural Science Foundation of China (31870928 and 32271360), Natural Science Foundation of Guangdong Province, China (2023A1515010829), Guangdong Provincial Basic and Applied Basic Research Fund Project (2025A1515220025) and the Blood Purification Plan of Guangdong Weiji Medical Development Foundation (No. K-20240103, K-202505-1-13 and K-202505-1-A). |
| 19 | **Data and data sharing** | Provide the data used to perform all analyses or report where and how the data can be accessed, and reference these sources in the article. Provide the statistical code needed to reproduce the results in the article, or report whether the code is publicly accessible and if so, where | 21 | All analysed datasets are all openly available in public databases. Specifically, the plasma proteomic datasets, GWAS summary statistics, transcriptomic, and single-cell transcriptomic data (GEO), as well as eQTL/sceQTL data, can be accessed through their respective public repositories (e.g., GWAS Catalog, GEO, eQTLGen, and OneK1K) as described in the Methods section. ELISA data generated in this study are available from the corresponding author on reasonable request. |
| 20 | **Conflicts of Interest** | All authors should declare all potential conflicts of interest | 20 | The authors declare that they have no known competing financial interests or personal relationships that could have appeared to influence the work reported in this paper. |

This checklist is copyrighted by the Equator Network under the Creative Commons Attribution 3.0 Unported (CC BY 3.0) license.

1.	Skrivankova VW, Richmond RC, Woolf BAR, Yarmolinsky J, Davies NM, Swanson SA, et al. Strengthening the Reporting of Observational Studies in Epidemiology using Mendelian Randomization (STROBE-MR) Statement. JAMA. 2021;under review.

2.	Skrivankova VW, Richmond RC, Woolf BAR, Davies NM, Swanson SA, VanderWeele TJ, et al. Strengthening the Reporting of Observational Studies in Epidemiology using Mendelian Randomisation (STROBE-MR): Explanation and Elaboration. BMJ. 2021;375:n2233.
